# Demonstration of a Gel-Polymer Electrolyte-Based Electrochromic Device Outperforming Its Solution-Type Counterpart in All Merits: Architectural Benefits of CeO_2_ Quantum Dot and Nanorods

**DOI:** 10.1021/acsami.3c16506

**Published:** 2024-01-19

**Authors:** Gaurav Kumar Silori, Subashchandrabose Thoka, Kuo-Chuan Ho

**Affiliations:** † Department of Chemical Engineering, National Taiwan University, Taipei 10617, Taiwan; ‡ Institute of Polymer Science and Engineering, National Taiwan University, Taipei 10617, Taiwan; § Advanced Research Center for Green Materials Science and Technology, National Taiwan University, Taipei 10617, Taiwan

**Keywords:** flexible electrochromic devices, gel-polymer electrolyte, CeO_2_ nanofillers, quantum dot and nanorod, stress−strain, haze analysis, oxygen vacancies, surface defects

## Abstract

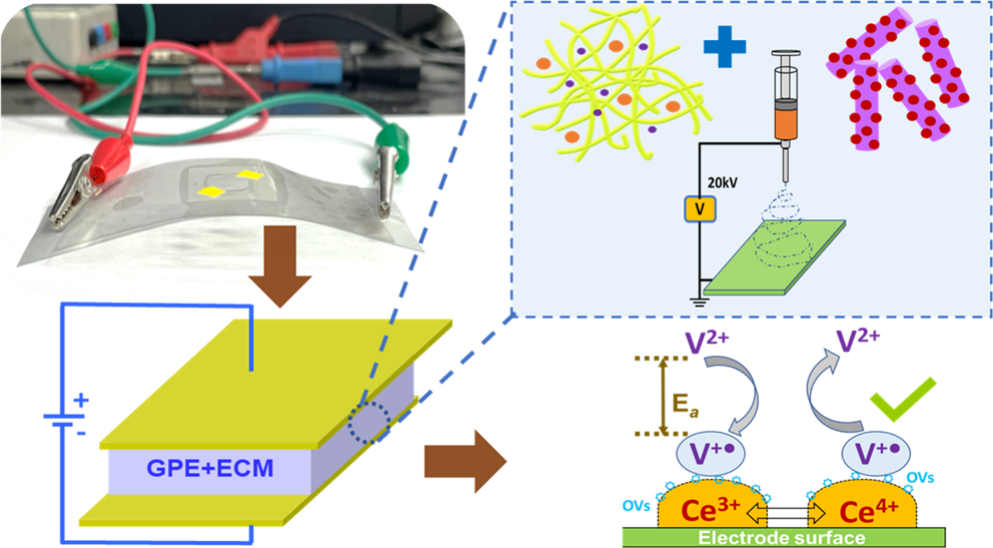

For years, solution-type electrochromic devices (ECDs) have intrigued researchers’ interest and eventually rendered themselves into commercialization. Regrettably, challenges such as electrolyte leakage, high flammability, and complicated edge-encapsulation processes limit their practical utilization, hence necessitating an efficient alternate. In this quest, although the concept of solid/gel-polymer electrolyte (SPE/GPE)-based ECDs settled some issues of solution-type ECDs, an array of problems like high operating voltage, sluggish response time, and poor cycling stability have paralyzed their commercial applicability. Herein, we demonstrate a choreographed-CeO_2_-nanofiller-doped GPE-based ECD outperforming its solution-type counterpart in all merits. The filler-incorporated polymer electrolyte assembly was meticulously weaved through the electrospinning method, and the resultant host was employed for immobilizing electrochromic viologen species. The filler engineering benefits conceived through the tuned shape of CeO_2_ nanorod and quantum dots, along with the excellent redox shuttling effect of Ce^3+^/Ce^4+^, synchronously yielded an outstanding class of GPE, which upon utilization in ECDs delivered impressive electrochromic properties. A combination of features possessed by a particular device (QD-NR/PVDF-HFP/IL/BzV-Fc ECD) such as exceptionally low driving voltage (0.9 V), high transmittance change (Δ*T*, ∼69%), fast response time (∼1.8 s), high coloration efficiency (∼339 cm^2^/C), and remarkable cycling stability (∼90% Δ*T*-retention after 25,000 cycles) showcased a striking potential in the yet-to-realize market of GPE-based ECDs. This study unveils the untapped potential of choreographed nanofillers that can promisingly drive GPE-based ECDs to the doorstep of commercialization.

## 1 Introduction

Indoor climate regulation remains one of the biggest energy consumption areas in modern society, with a 40% share of the total energy consumption.^1−3^ In the age of increasing climate challenges, the development of energy-saving materials and devices is highly desirable for sustainable development and the reduction of greenhouse emission. Electrochromism refers to a special class of materials whose optical properties could be reversibly adjusted by the application of induced potential.^4−6^ Any gadget utilizing electrochromic materials (ECMs) for an indoor/outdoor application could be referred to as an electrochromic device (ECD). Historically, ECDs have found applications in self-dimming rear mirrors for vehicles and in optical displays.^7,8^ Over the years, electrochromism has grown into a promising energy-saving technology. Their renewed interest could be found in electrochromic intelligent windows with tunable heat and light control features, thus emerging as a potential alternative to conventional windows.^9^ The ECD’s wide-ranging potential application scope includes energy-saving devices, aesthetic functions, electrochromic e-skins, and electrochromic papers.^10−13^ A recent commercial application of ECD was found in Boeing’s 787 Dreamliner airplane, where the manually operated conventional windows were replaced by electrochromic windows.^14^

Although electrochromic devices have been a research topic over the past five decades, their commercial applicability is still hindered by issues such as electrolyte leakage, electrolyte volatility, intricate edge-encapsulation process, and flammability in conventional liquid/solution-type ECDs.^15,16^ To address such issues, a new class of electrolyte known as solid polymer electrolyte (SPE) was utilized in the ECD configuration, and it has found growing research interest in the past decade.^17,18^ However, the poor ionic conductivity and sluggish response time of SPEs led researchers to seek a modification in the existing SPE configuration, thus paving the path for a new class of electrolytes known as gel-polymer electrolytes (GPEs). The typical configuration of a GPE consists of a host polymer matrix accommodating a liquid-phase electrolyte species.^19^ GPEs consist of features realized in liquid electrolytes, such as good ionic conductivity and superior electrode/electrolyte interaction, while maintaining the advantages of solid polymer electrolytes, such as good mechanical durability, high thermal endurance, and leak-proof configuration. A combination of additional advantages, such as the workability of polymers, adjustable shape, and stretchability allows GPEs to be potential candidates in portable and wearable electronic applications.^20,21^ However, despite the benefits mentioned above, conventional GPEs still suffer from low ionic conductivity, poor flame retardability (although far better than liquid electrolytes), and low mechanical endurance.^19,22,23^ To address this challenge, various inorganic/organic fillers have been incorporated into the GPE matrix, including nanofillers such as Al_2_O_3_, SiO_2_, TiO_2_, ZrO_2_, and BaTiO_3_.^24−26^ The filler’s induction has witnessed astounding improvement in electrochemical performance as well as in the physical strength of the GPE system, and the scientific attention is apparent in the unprecedented number of reports on applications in batteries,^27,28^ supercapacitors^29,30^ and dye-sensitized solar cells (DSSCs).^31,32^ Interestingly, several recent studies have revealed that the electrochemistry of GPEs strongly depends on the morphology, size, specific surface area, and exposed facets/planes of utilized nanofillers.^33−36^ This realization has motivated researchers to delve into the marvels of the nanoworld by creating new and novel nanostructures, thus unfolding a new and exciting field of filler engineering.

While the quest for effective nanofillers has surged enormously, interestingly, in a parallel realm, CeO_2_-based nanomaterials have gained rampant interest in applications such as photocatalytic reactions, solid oxide fuel cells (SOFCs), and three-way catalysis (TWC) because of their higher oxygen storage capacity (OSC), available defect surfaces, and unique ability to shuttle between Ce^4+^ and Ce^3+^ redox states.^37,38^ For instance, Baldim et al. evaluated the enzyme-like catalytic activity in a series of CeO_2_ nanoparticles with particle sizes of ∼5, ∼8, 23, and 28 nm. The result showed that smaller quantum dot particles enhanced the catalytic effect due to the proliferated Ce^3+^ fraction (14–43%) compared to the larger counterparts (Ce^3+^: 9–10%).^39^ Spezzati and co-workers, in a recent report, analyzed CO oxidation by Pd supported on CeO_2_ nanorods (exposing 111 facets) and nanocubes (exposing 100 facets). The nanorod-supported structure demonstrated a higher oxidation rate (1 × 10^21^ molecules CO g_Pd_^–1^ s^–1^) than the nanocube-supported nanostructure (3 × 10^20^ molecules CO g_Pd_^–1^ s^–1^) at 50 °C. The reasons were traced from density functional theory (DFT) calculations, which showed that (111) facets involve a lower free energy barrier compared to (100) facets.^40^ Shen’s group studied the effect of CO oxidation with three different morphologies of CeO_2_ (nanowires, nanorods, and nanoparticles) and observed that rod-shaped CeO_2_ nanostructure delivered high CO oxidation due to the availability of reactive planes (110 and 100).^41^ In addition to the above-mentioned reports, some excellent reviews have also shed light on the vast applicability of CeO_2_ nanostructures.^37,38,42^ However, despite the established distinctive advantages, scarce reports in the literature suggest that nanofillers’ applicability in ECD applications has still not found necessary attention, thus posing a significant scope for researchers interested in the field. It is a pressing need that developments made in the field-specific domain are equally embraced by coexisting research areas so as to comply with state-of-the-art trends. An extensive literature search suggests that CeO_2_ fillers in GPE assemblies have never been conceived for ECD applications.

Thus, in pursuit of our interest, the notion of the conceived study was exercised by utilizing nanoengineered CeO_2_ fillers for the development of a novel set of GPEs—later to be employed in ECDs for improved electrochromic and switching performance. For GPE processing, herein, poly(vinylidene fluoride-*co*-hexafluoropropylene) or PVDF-HFP was chosen as a host polymer due to its ability to render fast segmental motion of the polymer chain, low glass-transition temperature, and wide electrochemical window.^43^ The imidazolium-based ionic liquid (IL)BMIMBF_4_ was utilized as a salt transporter due to the low dissociation energy for easier ion mobilization and enhanced solvating properties.^44^ The organic solvent propylene carbonate (PC) was added to the polymer matrix as a plasticizer for the increased dissociation of ions and electrochemical stability.^45^ For CeO_2_ nanofillers, initially, two types of filler shapes were synthesized, i.e., zero-dimensional (0D) quantum dots (∼7 nm) and one-dimensional (1D) nanorods (∼50 nm). The quantum size adoption for CeO_2_ fillers in our study derived motivation from recent reports highlighting fillers’ remarkable performance upon their utilization as quantum dots.^46,47^ Further, the nanorod shape was chosen for its anisotropic ability combined with the superior electrical or mechanical properties required in a nanocomposite matrix.^48,49^ A novel hybrid filler structure was also conceived and synthesized with quantum dots (QDs) *in situ* grown over nanorod (NR) surfaces. This QD-NR nanostructure was hypothesized to not only hinder aggregation of QDs but also create more edge and surface defects, induce oxygen surface vacancies and, thus, enhance redox-active centers. The polymer electrolyte was nanoweaved through the electrospinning technique due to the latter’s structural engineering qualities, which stems from its versatility to create flexible nanofibers, porous fiber morphologies, large specific surface area, and surface functionality.

## 2 Experimental Section

### 2.1 Materials and Reagents

Cerium(III) nitrate hexahydrate (≥99.9%, Alfa Aesar), ammonium hydroxide (28–30%, J.T. Baker), acetone (≥99.5%, J.T. Baker), poly(vinylidene fluoride-*co*-hexafluoropropylene) (PVDF-HFP; Sigma-Aldrich), sodium hydroxide (≥97.0%, Sigma-Aldrich), 1-butyl-3-methylimidazolium tetrafluoroborate (BMIMBF_4_; ≥98.0%, Sigma-Aldrich), and *N*,*N*-dimethylformamide (DMF; ≥99.8%, Macron Fine Chemicals) were long-placed in a vacuum before use. Reagents for the preparation of the electrolyte solution were used as received and include tetrabutylammonium tetrafluoroborate (TBABF_4_; 99%, Sigma-Aldrich), sodium tetrafluoroborate (NaBF_4_; 98%, Sigma-Aldrich), propylene carbonate (PC; 99%, Alfa Aesar), and ferrocene (Fc; >98%, TCI Co.). As-obtained benzyl viologen dichloride (BzVCl_2_; 99%, Sigma-Aldrich) was used as a precursor for the synthesis of electrochromic species. Pure water of 18.2 MΩ·cm at 25 °C (Merck Millipore SIMSVO1JP) was utilized for synthesis purposes and elsewhere. Indium tin oxide (ITO) coated on glass (7 Ω/sq., Solaronix SA Inc.) and PET (40 Ω/sq., Southwall Europe GmbH) substrate were utilized as a terminal electrode during the ECD fabrication.

### 2.2 Synthesis Procedure of Nanofillers and the Electrochromic Material

The synthesis method of CeO_2_ quantum dots^50^ and CeO_2_ nanorods^51^ was based on recent reports. An *in situ* synthesis method was developed for the synthesis of CeO_2_ quantum dots anchored over nanorod surfaces (labeled as QD-NR). In brief, 1.70 g of Ce(NO_3_)_2_·6H_2_O and 50 mg of CeO_2_ nanorods were dissolved in 50 mL of distilled water to obtain a homogeneous mixture. After 10 min, 2.5 mL of NH_4_OH (0.5 M) and 7.5 mL of NaOH (0.1 M) were added dropwise to the prepared homogeneous mixture and further stirred for 15 min. The resultant solution was then placed in a 100 mL autoclave tube (Teflon-lined stainless steel) for the *in situ* growth of the desired nanostructure during hydrothermal treatment (140 °C for 1 h). After cooling to room temperature, the obtained solution was washed multiple times with DIW and EtOH at 20,000 rpm. Lastly, the synthesized QD-NR particles were filtered and vacuum-dried for further use. The synthesis process of the utilized electrochromic material (benzyl viologen, BzV) was based on our previous report.^52^

### 2.3 Preparation of Filler-Incorporated Polymer Electrolyte

PVDF-HFP (0.750 g) pellets were dissolved in 3.250 g of DMF/acetone (v/v 60:40) and stirred overnight at 70 °C to obtain a homogeneous polymeric solution. Meanwhile, 15 wt % BMIMBF_4_ (112.5 mg, PVDF-HFP basis) and designated concentrations of synthesized CeO_2_ nanofillers (0.5, 1.0, 1.5, and 2.0 wt %; 3.75, 7.50, 11.25, and 14.00 mg) were separately dissolved in 1.0 g of DMF/acetone (60/40) solution through sonication. The nanofiller-dispersed solution was then added dropwise to the earlier-prepared PVDF-HFP polymeric solution. The filler-doped polymeric solution was first stirred for 60 min at room temperature, followed by ultrasonication for 120 min to ensure homogeneous mixing. The resultant solution was processed using electrospinning (20 kV, 0.01 mL/min) for 20 min, adjusting the injector and substrate-collector distance at 15 cm. The obtained electrospun nanofiber mats (electrolyte host) were vacuum-dried at 60 °C overnight.

### 2.4 Engineering of Filler-GPE-Based ECDs

Initially, the ITO-1 substrate was drilled into two diagonal holes for ease of solution injection, followed by isopropanol wiping and 20 min treatment with UV ozone (UVO CLEANER Model 42, Jelight Company Inc.). The electrospun nanofiber film obtained on the ITO-2 substrate (3.0 × 2.0 cm^2^) was carefully tailored to a size of 1.0 × 1.0 cm^2^. Afterward, the conductive parts of ITO-1 and ITO-2 were firmly sandwiched and heat-laminated (through the DuPont 60 μm Surlyn frame) to achieve a 1.0 × 1.0 cm^2^ active area of the host polymer electrolyte. The polymer electrolyte was further injected (through drilled holes on ITO-1) with a plasticizer mixture of 0.05 M benzyl viologen (BzV), 0.05 M ferrocene (Fc), and 0.5 M TBABF_4_ in PC to complete the fabrication of ECD. The white electrolyte membrane immediately turns into a transparent film upon the injection of the plasticizer mixture. In this study, five kinds of ECD configurations were prepared: three GPE-based ECDs with different varieties of CeO_2_ nanofillers, a GPE-based ECD without filler (PVDF-HFP/IL/BzV-Fc ECD), and a solution-type ECD (LEP/BzV-Fc ECD). The filler-GPE-based ECDs were labeled based on the nanofiller types incorporated within: devices with nanorod (NR), quantum dot (QD), and hybrid QD-NR fillers as NR/PVDF-HFP/IL/BzV-Fc ECD, QD/PVDF-HFP/IL/BzV-Fc ECD, and QD-NR/PVDF-HFP/IL/BzV-Fc ECD, respectively. A similar procedure was followed to fabricate flexible ECDs by replacing the rigid glass substrates with flexible PET/ITO ones.

### 2.5 Material Characterization and Measurements

Scanning electron microscopy (SEM/FE-SEM; NOVA NanoSEM 230, Thermo Fisher Scientific) was employed to analyze the electrospun nanofiber matrix. Transmission electron microscopy (TEM/HR-TEM; FEI Tecnai F30 G2, 300 kV) was performed for the shape, size, and lattice evolutions of nanofillers. X-ray diffraction (XRD; Rigaku Smart Lab SE.) analysis was carried out to ensure the successful synthesis of nanofillers. For XRD, a scattered angle (2θ) recording in the 10–90° range was employed at a scan rate of 5° min^–1^. X-ray photoelectron spectroscopy (XPS; Al Kα X-ray source, Thermo Fisher Scientific Inc., Waltham, U.K.) analysis of utilized fillers was carried out at ultrahigh vacuum (1 × 10^–9^ mbar) for surface chemistry and electronic state information. For probing defects and oxygen vacancies, the electron paramagnetic resonance (EPR) test of nanofillers was carried out through a Bruker EMXmicro spectrometer at a frequency of 9.838 GHz and microwave power of 34.5 mW. The spectra were recorded at room temperature. The tensile strength of the electrospun polymer electrolyte (with and without fillers) was performed on an electromechanical test system (MTS Criterion Model 42). The polymer membranes carried out for the testing were prepared by electrospinning the filler-incorporated polymer solution for 150 min (20 kV, 0.01 mL/min). The obtained membranes (thickness, 25–28 μm) were vacuum-dried overnight at 70 °C and afterward tailored into 5 cm × 2 cm sizes for the stress–strain testing. The test was performed by adjusting the electrolyte membrane in the designated clamps and enabling a 250 N load cell at the crosshead speed of 10 mm min^–1^.

### 2.6 ECD Characterization

The cyclic voltammetry (CV) and electrochemical impedance spectroscopy (EIS) analysis of the ECDs were carried out through an electrochemical workstation (Autolab PGSTAT302N, Metrohm). The deuterium and halogen light source (DH-2000-BAL) enabled spectrometer (Ocean USB4000 Fiber Optic) was employed to measure the optical properties, such as absorbance and transmittance of the fabricated ECDs. The thermal imaging of fabricated ECDs was carried out through an IR-enabled camera recorder (Fluke TiS75+). The photostability of the ECDs was analyzed through a solar simulator (Thermal Oriel 1000 W) abetted with a xenon lamp at AM 1.5G spectrum (100 mW cm^–2^). A haze meter (NDH 2000, Nippon Denshoku, Tokyo, Japan) was utilized to ascertain the levels of haze in the assembled ECDs.

## 3 Results and Discussion

### 3.1 XRD and XPS Analyses of As-Synthesized Nanofillers

**Figure 1:**
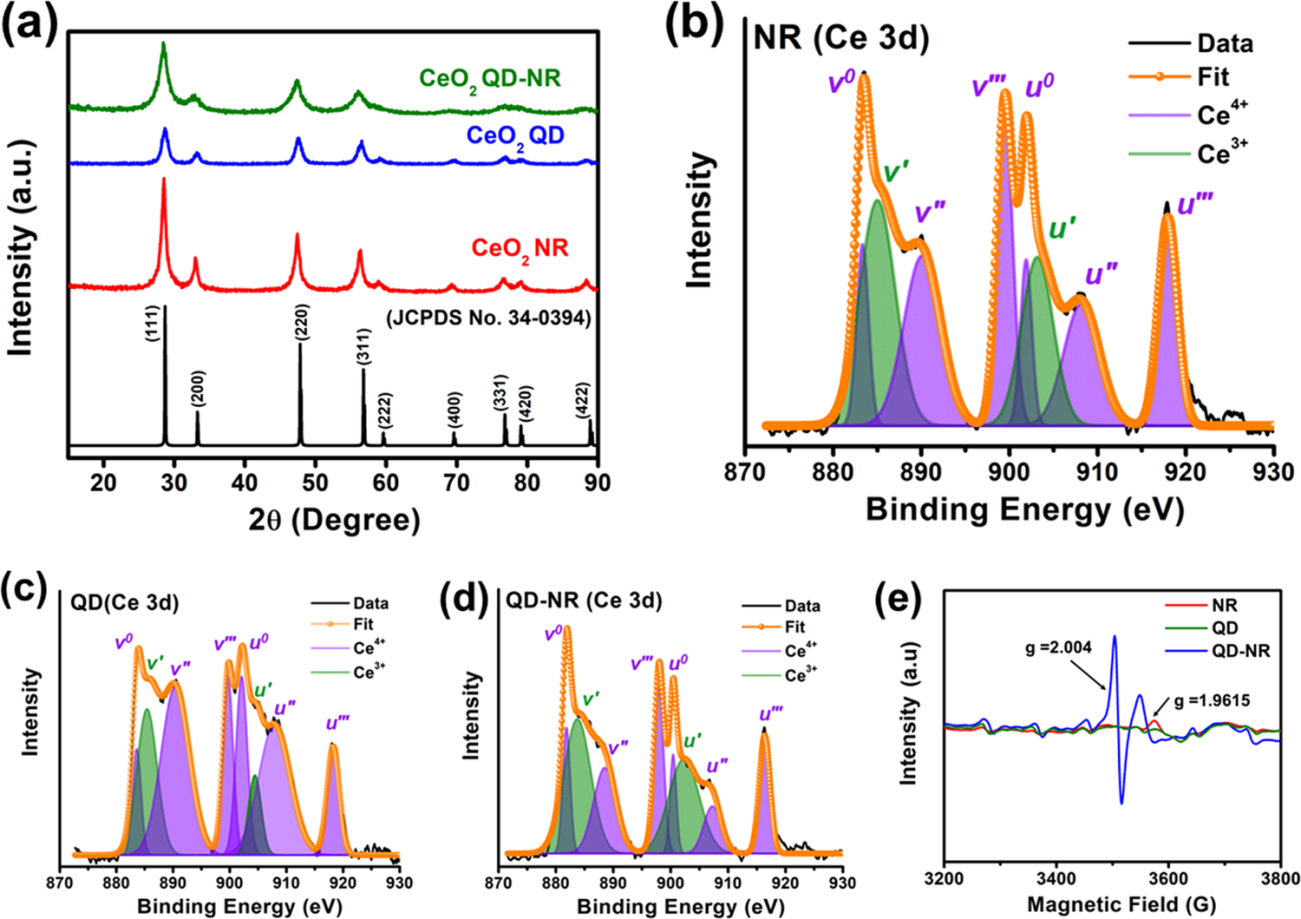
(a) XRD spectra of as-synthesized CeO_2_ (NR, QD, and QD-NR) nanofillers. Ce 3d XPS spectra of CeO_2_ (b) NR, (c) QD, and (d) QD-NR. (e) EPR spectra of CeO_2_ NR, QD, and QD-NR.

The obtained XRD spectra of the as-synthesized fillers were indexed to the conventional structure of CeO_2_ (face-centered cubic, JCPDS card no: 34-0394), as shown in Figure 1a. The XRD pattern for the nanorod crystal in Figure 1a revealed a relatively high presence of stable (111) surfaces in comparison to reactive (200) and (220) planes. In contrast, the surface of quantum dot nanofillers showed an almost equal distribution of nonreactive (111) and reactive (220) planes, along with the small presence of (200) planes. The decreased number of stable (111) surfaces in CeO_2_ QDs is speculated to be due to lattice expansion usually seen with the decreased size of ceria particles.^53^ The hybrid structure of QD-NR exhibited sufficient (111) planes due to the presence of NR with considerable availability of reactive (220) and (311) surfaces. Noticeably, the 2θ peaks at ∼28° (111) and ∼33° (200) slightly merged and broadened for QD-NR nanostructures; the same phenomena were also observed at ∼77° (331) and ∼79° (420). In general, all 2θ peaks of QD-NR displayed some broadening in comparison to NR and QD counterparts. The broadening of peaks here indicates the effect endowed from microstrains, such as the creation of defects, dislocations, and prevailed stress within the crystal lattice. Further, the presence of 200 and 220 planes suggests the availability of exposed 100 and 110 facets, respectively, on the nanofiller’s surfaces. Recent studies are in good agreement that the (100) and (110) facets of CeO_2_ nanostructures are more reducible and active due to the less energy required for oxygen vacancy generation; briefly, in terms of energy requirement, (110) < (100) < (111).

The XPS spectra of the as-synthesized nanofillers along with simulated peaks are shown in Figure 1b–d. The obtained Ce 3d spectrum of synthesized nanofillers can be deconvoluted into eight peaks labeled as v^0^, v′, v″, v‴, u^0^, u′, u″, and u‴, where v and u correspond to spin–orbital multiples 3d_3/2_ and 3d_5/2_, respectively. In addition, the peaks appearing as v^0^, v″, v‴, u^0^, u″, and u‴ at ∼882, ∼890, ∼899, ∼903, ∼907, and ∼917 eV correspond to Ce^4+^, while v′ and u′ at ∼884 and ∼904 eV are assigned to Ce^3+^ states. The reversible shuttling of Ce^4+^ ↔ Ce^3+^ redox couples is commonly associated with the localization/delocalization of 4f electrons wherein oxygen vacancies are generated through the hopping mechanism.^54^ For quantitative evaluation, the estimation of Ce^3+^ and Ce^4+^ redox centers was carried out by their corresponding peak areas in accordance with the following formulas^55^


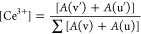
1
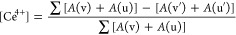
2

The contents of Ce^3+^ and Ce^4+^ redox centers are calculated and tabulated in Table S1 of the Supporting Information. The Ce^3+^ contents for NR, QD, and QD-NR were calculated to be 26.7, 19.7, and 32.2%, respectively, indicating the enriched transition of the Ce^4+^ ↔ Ce^3+^ couple for NR and QD-NR nanostructures. The relatively lower Ce^3+^ content for QDs possibly lies in the aggregation affinity of QDs, thus mitigating the Ce^4+^ ↔ Ce^3+^ transition sites. The increased Ce^3+^ redox centers for QD-NR are believed to arise from retrieved active surfaces due to structural engineering benefits, the proliferation of (110) and (100) facets, and defect junctions created at the QD/NR interface.

The EPR spectra recorded for the synthesized CeO_2_ nanofillers are highlighted in Figure 1e. Any defect, free radicals, or other paramagnetic centers in a tested species may be reliably and sensitively identified by the EPR test. As seen in Figure 1e, the EPR for NR and QD represented a weak signal at room temperature. The unavailability of defect junctions and the majority of the Ce^4+^ ions in the stable state (111 planes), which corresponds to an electronic configuration of [Xe] 4f^0^ 5d^0^ 6s^0^, can be attributed to the diminished EPR signal shown by the NR and QD fillers.^56^ However, miniature peaks at around *g* ∼ 1.9615 were observed for the NR and QD fillers, which suggests the presence of bulk Ce^3+^ states. In contrast, a strong symmetric peak at around *g* ∼ 2.004 was registered for QD-NR fillers, which is an indication of rich Ce^3+^ with numerous surface oxygen vacancies created through induced microstrains in the hybrid structure. From the recorded data, it is inferred that the morphology and shape of nanofillers have a noticeable effect on the EPR signal intensity. The results obtained from the EPR analysis are in good agreement with the XPS analysis.

### 3.2 Morphology Analysis of As-Synthesized Nanofillers

**Figure 2:**
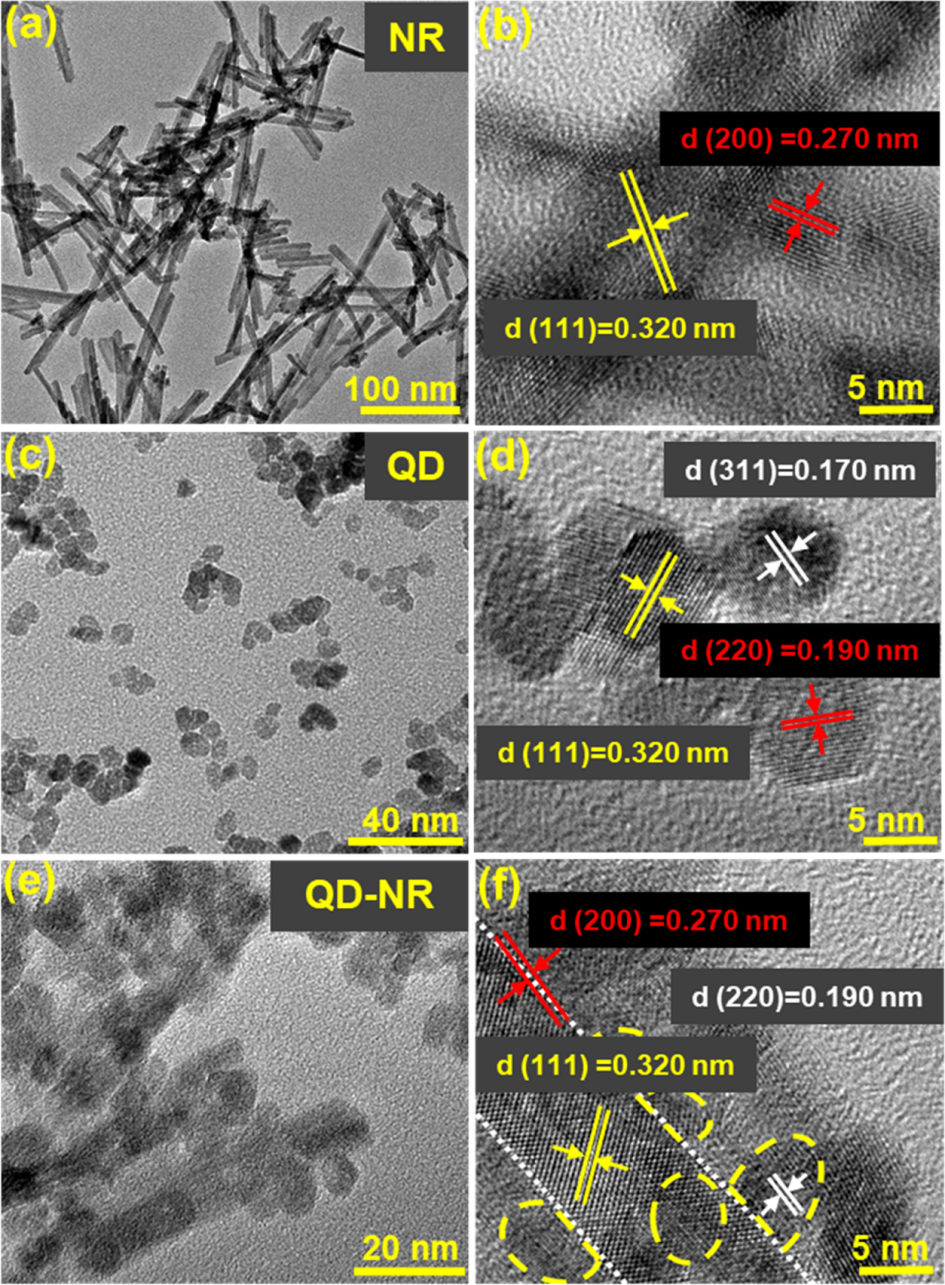
TEM images of CeO_2_ (a) NR, (c) QD, and (e) QD-NR. HR-TEM images of CeO_2_ (b) NR, (d) QD, and (f) QD-NR.

The TEM and HR-TEM images of the as-synthesized nanofillers are shown in Figure 2a–f. The interplanar spacings or *d*-spacings were extracted from HR-TEM images using a digital micrography tool (GMS 3). It is evident from Figure 2a,c that nanorods and quantum dots in ∼50 and ∼7 nm sizes were successfully synthesized. Further, a closer HR-TEM view (Figure 2b) showed interplanar spacings of 0.320 and 0.270 nm on the nanorod stem, indicating the major presence of (111) and (200) lattice fringes, respectively. The QD surface realized *d*-spacings of 0.190 and 0.170 nm, indicating the readiness of (220) and (311) lattice fringes, respectively (Figure 2d). Apart from the dominantly exposed lattice spacing of 0.320 nm, the QD-NR filler structure demonstrated enough interplanar spacings of 0.270 and 0.190 nm (Figure 2f), particularly near the QD/NR interface, which likely appears due to microstrains and corrugations.

The interplanar spacing revealed through HR-TEM analysis agrees well with the information on exposed planes obtained through XRD analysis.

### 3.3 FE-SEM Analysis of Electrospun Nanofibers

**Figure 3:**
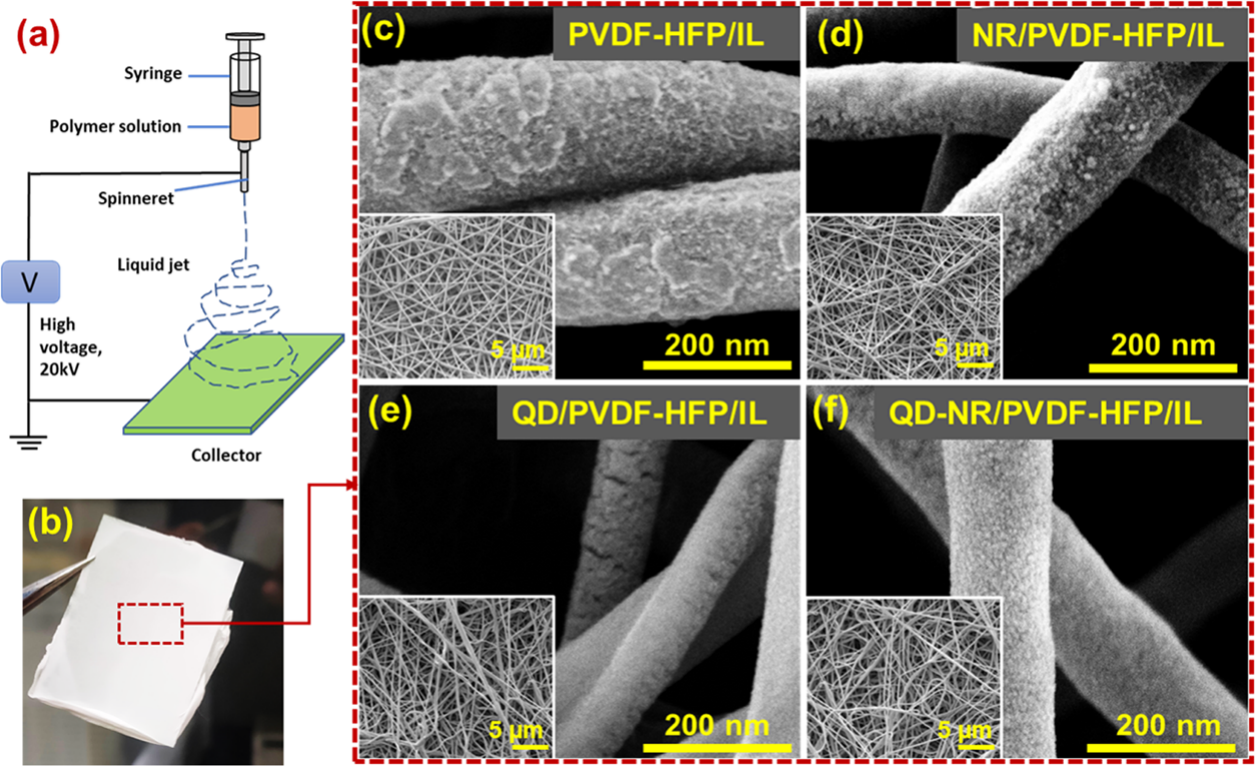
(a) Schematic of electrospinning instrumentation. (b) Representative image of the electrospun membrane host. FE-SEM images of (c) electrospun PVDF-HFP/IL, (d) loaded with NR, (e) QD, and (f) QD-NR nanofillers; inset SEM images show the corresponding fiber network in a distant view.

The schematic for the utilized electrospinning setup and the resultant electrospun membrane is shown in Figure 3a and b, respectively, while the FE-SEM images of as-spun nanofibers, with and without fillers, are shown in Figure 3c–f. It is noteworthy to mention that an optimized filler loading was first determined (1 wt %, PVDF-HFP basis) for the incorporation in the precursor polymer solution, and the basis for this selection is discussed in the Supporting Information (Method S1.1, Figure S2, and Table S2) of this work. As shown in Figure 3c, the electrospinning process without filler incorporation produced smooth-surfaced nanofibers (PVDF-HFP/IL) with an average diameter of 150–200 nm. The inset images in Figure 3c–f demonstrate a well-structured morphology of the fiber network suitable for the entrapment of liquid electrolytes. Further, the incorporation of fillers in the fiber network resulted in a nanofiber stem partially (NR/PVDF-HFP/IL) or totally covered (QD/PVDF-HFP/IL and QD-NR/PVDF-HFP/IL) with filler loading as seen in Figure 3d–f. The filler-loaded nanofibers’ average diameter ranged from 100 to 150 nm, and this slight decrement in diameter as compared to the filler-less counterpart originates from the reduced viscosity of the polymeric solution due to the filler’s incorporation, thus producing a thinner nanofiber in the electrospinning process. Importantly, no accumulation or bed formation was observed (inset images) upon the filler’s induction, mainly due to optimized nanofiller loading, a desirable feature for better mobility of ions. It is apparent from Figure 3d–f that filler loading on the fiber stem caused the generation of several cracked rings and cavity-like-formations, which might favor enhanced entrapment of the liquid electrolyte with the added benefit of defect surfaces, thus producing more active sites for interactions.

### 3.4 Porosity, Electrolyte Uptake, and Degradation Tendency of Electrospun Membranes

**Figure 4:**
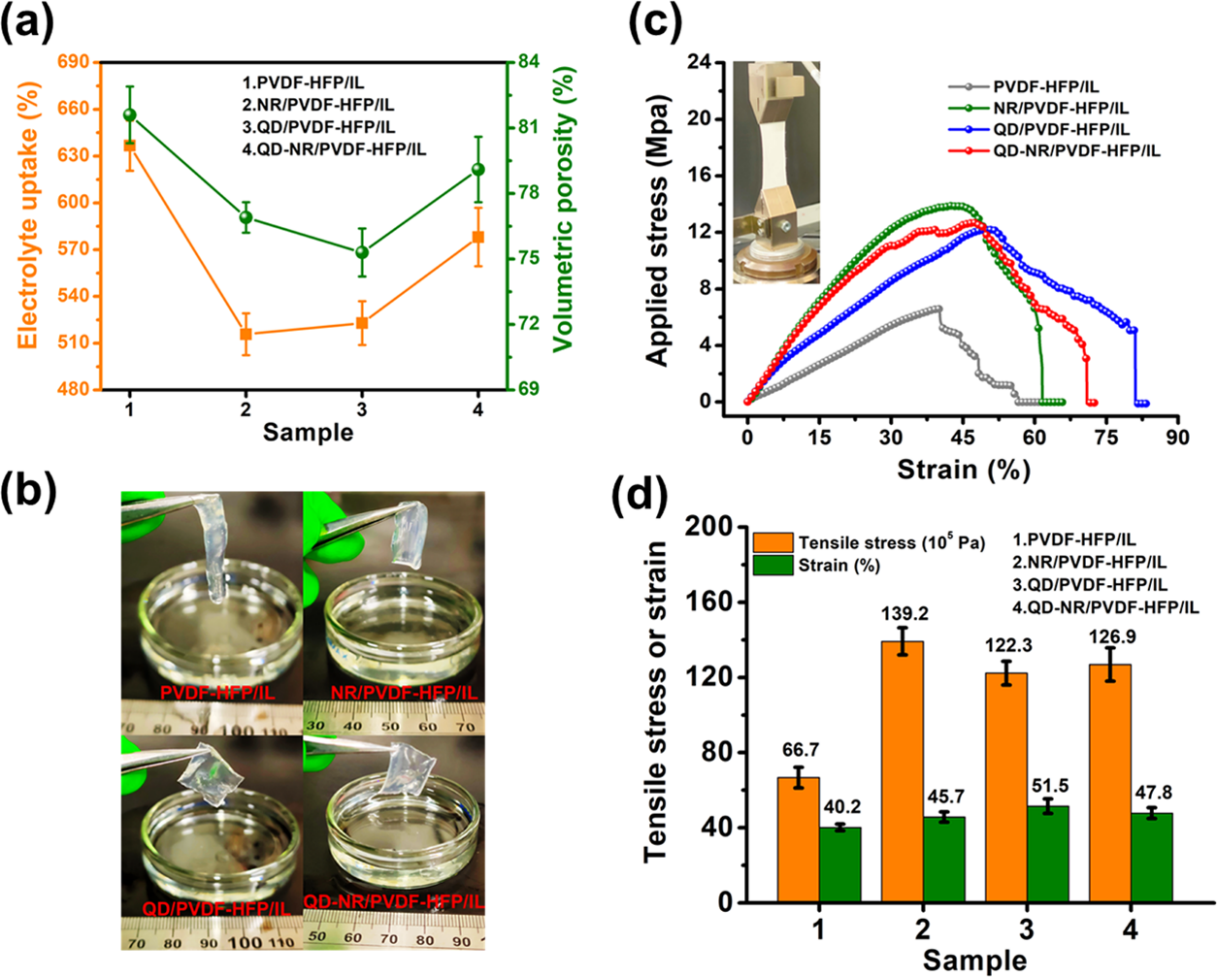
(a) Electrolyte uptake (%) and volumetric porosity (%) plot of electrospun PVDF-HFP/BMIMBF_4_ membranes at 25 °C. (b) Images of CeO_2_ filler-based electrospun PVDF-HFP/BMIMBF_4_ membranes for 48 h in an electrolyte media of 0.5 M TBABF_4_ in PC. (c) Stress (MPa) vs strain (%) curves, (d) tensile stress (10^5^ Pa) and strain (%) quantification of pristine and CeO_2_ filler-based electrospun PVDF-HFP/BMIMBF_4_ membranes.

The membrane characterization, such as electrolyte uptake (EU) and volumetric porosity (VP), can help understand the structural benefits achieved in the nanofiber-based electrolyte assembly. In this regard, the EU of as-spun membranes was determined at 25 °C through complete immersion of the dry polymeric membranes in a liquid-electrolyte solution (0.5 M TBABF_4_ in PC) for 15 min. After electrolyte entrapment, the membranes were removed from the solution and weighed; before weighing, excess electrolytes from the membranes were removed by carefully placing a mild glass weight (∼30 g). The EU of the as-spun membranes was calculated from the following widely accepted formula^57^
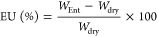
3where *W*_dry_ is the weight of dry as-spun polymeric membranes and *W*_Ent_ is the membrane weight after liquid-electrolyte entrapment. Further, the volumetric porosity of the obtained nanofiber membranes was calculated from the following equation^58^
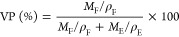
4

where *M*_F_ and *M*_E_ are masses and ρ_F_ and ρ_E_ are densities of the electrospun nanofiber (here PVDF-HFP) and the entrapped electrolyte (PC), respectively. The calculated EU and VP of polymeric membranes are tabulated and plotted in Table S3 and Figure 4a, respectively. The graph shown in Figure 4a suggests that electrolyte uptake decreases with decreased porosity. The highest electrolyte uptake and porosity (∼636.7 and ∼81.6%) behavior of filler-less GPE (PVDF-HFP/IL) was rooted in an effective entrapment of the liquid electrolyte along with uncontrolled swelling issue encountered (Figure 4b) due to lack of mechanical strength. Gradual increases in EU (∼515.8, ∼522.9, ∼578.1%) and VP (∼75.3, ∼76.9, ∼79.1%) were seen for NR, QD, and QD-NR filler-based GPEs (NR/PVDF-HFP/IL, QD/PVDF-HFP/IL, and QD-NR/PVDF-HFP/IL, respectively), which is due to the geometric benefits provided by the filler structure in terms of less aggregation and generation of host cavities as seen in Figure 3d–f, thus hosting excess electrolytes. The degradation tendency of the electrospun polymer electrolytes was also tested by immersing the filler-less and filler-incorporated electrospun membranes in an electrolyte solution of 0.5 M TBABF_4_ in PC for an elongated time of 48 h. The membranes’ visuals before and after the electrolyte immersion are shown in Figure 4b. It was noticed that the filler-less PVDF-HFP/IL polymer film could not withstand its original geometry and degraded abruptly when exposed to the electrolyte solution, as seen in Figure 4b. In contrast, filler-incorporated GPEs maintained their structure well and did not deform even after 48 h; particularly, the QD/PVDF-HFP/IL and QD-NR/PVDF-HFP/IL polymer membranes showed improved mechanical and physical strength, as reflected in their well-retained shape.

### 3.5 Stress–Strain Analysis of Electrospun Nanocomposites

Reinforced mechanical properties of GPEs are crucial to guarantee physical endurance during the rigorous fabrication process and application in the state-of-the-art market of flexible electronics. Figure 4c demonstrates the stress–strain curves obtained for polymer membranes (with and without fillers), whereas Figure 4d quantifies and compares extracted data from Figure 4c. As is apparent, the tensile stress of composite membranes increased with the incorporation of CeO_2_ nanofillers; from ∼6.7 for filler-less composites to ∼13.9, ∼12.2, and ∼12.7 MPa for NR, QDs, and QD-NR-based composites, respectively. Also, the composite membranes witnessed an extension from the least strain of ∼40.2% (PVDF-HFP/IL) to an increased elongation of ∼45.7% (NR/PVDF-HFP/IL), ∼51.5% (QD/PVDF-HFP/IL), and ∼47.5% (QD-NR/PVDF-HFP/IL) upon filler incorporation. The reinforced stress and elongation behavior in filler-based membranes stems from the better interlocking of the polymer matrix due to arbitrarily created morphologies on the nanofiber stem, improved surface roughness due to filler loading, and enhanced interfacial stress transfer.^59−61^ The performed characterization evidenced that the synthesized morphologies of nanofillers deliver different degrees of electrolyte uptake, porosity, and mechanical strength to the polymer nanocomposite. This feature can be attributed to local polymer–nanofiller interactions, system modulus and viscoelasticity, permittivity gradient established by the filler–polymer interface, and dynamics of polymer chains caused by individual filler morphologies. Theoretical and experimental studies are in good agreement that the morphological variations of nanofillers might cause significant alteration to the physical properties of the nanocomposites.

### 3.6 Electrochemical and Optical Analyses of ECDs

#### 3.6.1 Impedance and Ionic Conductivity Analysis

**Figure 5:**
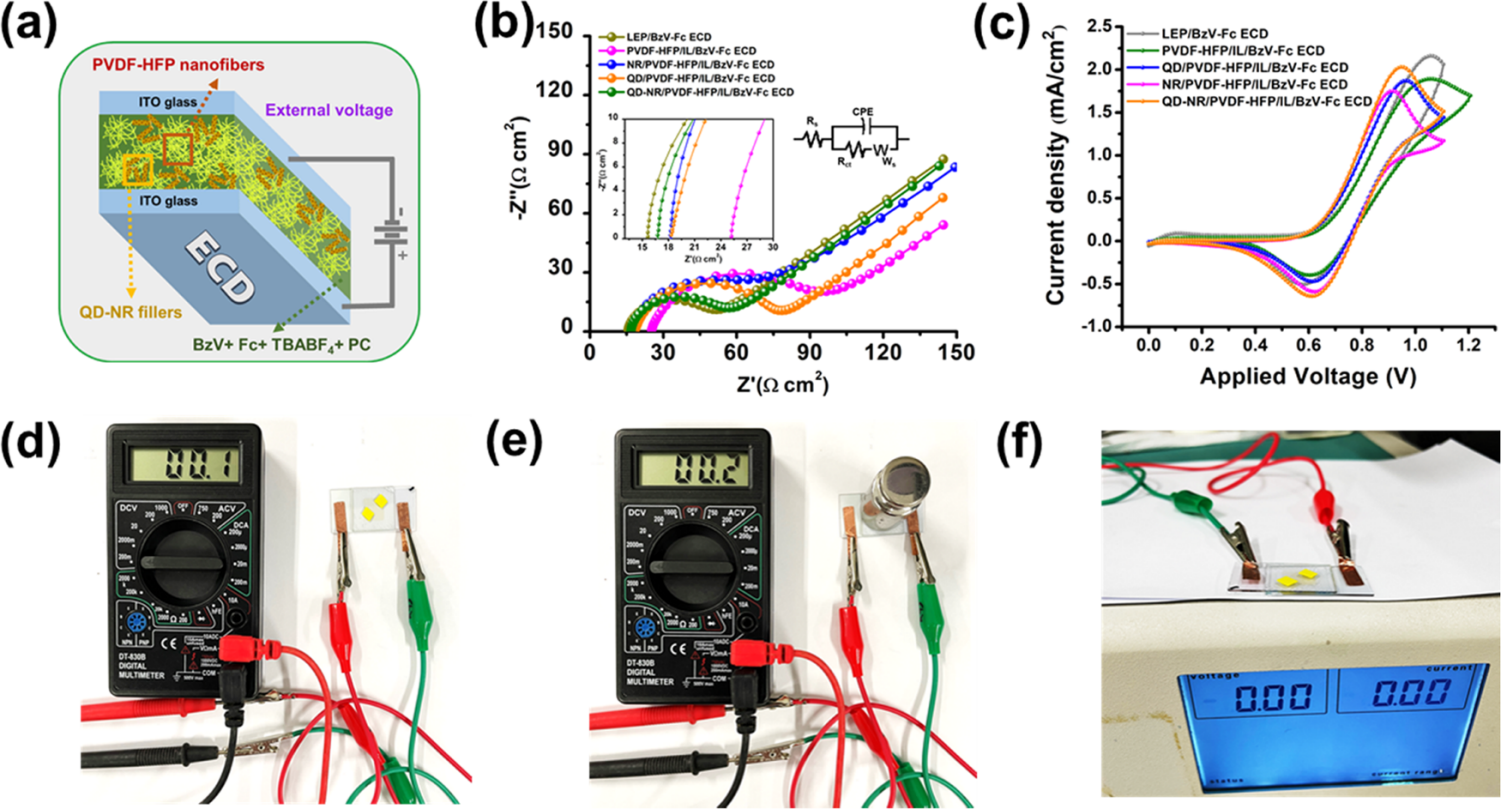
(a) Schematic of a QD-NR/PVDF-HFP/IL/BzV-Fc ECD. (b) Nyquist plots of pristine and CeO_2_ filler-based ECDs obtained through an AC stimulus of 10 mV in a frequency spectrum of 100 kHz to 0.1 Hz. (c) CV plots of solution-type LEP/BzV-Fc ECD and GPE-based ECDs (with or without CeO_2_ nanofillers); at a scan rate of 100 mV/s, the ECDs were scanned in the voltage range between 0 and 1.25 V. The open-circuit voltage (OCV) of QD-NR/PVDF-HFP/BzV-Fc ECD in (d) open and (e) under ∼200 g weight-press condition. (f) OCV recorded for QD-NR/PVDF-HFP/BzV-Fc ECD after 1000 CV cycles scanned between 0.0 and 1.1 V at a scan rate of 100 mV/s.

The assembly of a model ECD (QD-HR filler-based) used in this work is shown in Figure 5a. Electrochemical impedance spectroscopy (EIS), for the insight into impedance prevailing in the bulk electrolyte as well as at the electrode/electrolyte interface, was employed on ECDs by an AC stimulus of 10 mV in the frequency range of 100 kHz to 0.1 Hz. The obtained Nyquist plots (Figure 5b) were simulated through a software tool (ZSimpWin 3.20) and endowed with an equivalent circuit model with relatively smaller values (∼10^–4^) of chi-square (χ^2^), thus indicating a good fitting behavior. The inset image in Figure 5b shows the real-axis contact of the Nyquist plot, which contributes to resistance imparted by the bulk electrolyte, also known as solution resistance (*R*_s_). Further, the width of the semicircles governs the contribution from the electrode charge transfer resistance (*R*_ct_), wherein the following straight-line part is attributed to mass transport through diffusion, also known as the Warburg diffusion element (*W*_s_). The constant phase element (CPE) in an equivalent circuit model accounts for double-layer capacitance and its deviation from ideality. The parameter values of *R*_s_, *R*_ct_, and χ^2^ from the simulated Nyquist plots are presented in Table 1. The extracted values displayed an increment in *R*_s_ and *R*_ct_ upon transition from solution-based LEP/BzV-Fc ECD (∼15.6 and ∼44.7 Ω cm^2^) to GPE-based PVDF-HFP/IL/BzV-Fc ECD (∼25.2 and ∼92.8 Ω cm^2^); the *R*_s_ increment is majorly due to the transport barrier arising from the viscous GPE phase in comparison to the highly mobile liquid-electrolyte counterpart, whereas increased *R*_ct_ is likely due to the poor electrode–electrolyte adhesion stemming from the fast deformation tendency of PVDF-HFP/IL, as seen in Figure 4b. Clearly, the incorporation of CeO_2_ nanofillers in the ECD assembly witnessed a reduction in the impedance behavior as seen from smaller *R*_s_ and *R*_ct_ values of Table 1. The NR/PVDF-HFP/IL/BzV-Fc ECD and QD/PVDF-HFP/IL/BzV-Fc ECD ECDs displayed almost similar *R*_s_ (∼18.3 and ∼18.2 Ω cm^2^) and *R*_ct_ (∼73.2 and ∼71.0 Ω cm^2^) impedance, slightly bettered by the latter. The suppression in *R*_s_ impedance is assisted by the diminished viscosity of the polymer electrolyte upon an optimized loading of nanofillers.^62^ Also, the enhancement in charge transfer capability, in comparison to filler-less GPE, can be attributed to the filler morphology, rendering more active redox centers (Table S1) and thus enhanced interaction at the electrode/electrolyte interface. The QD-NR/PVDF-HFP/IL/BzV-Fc ECD almost approached the solution-based LEP/BzV-Fc ECD behavior (Table 1), with *R*_s_ and *R*_ct_ values of ∼16.7 and ∼48.8 Ω cm^2^, respectively; the enhancement is substantiated by the advantages realized in QD-NR-based GPE with improved electrolyte uptake and porosity as displayed in Figure 4a.

**Table 1:** Series Resistance, Charge Transfer Resistance, and Ionic Conductivities Obtained from the EIS Analysis of ECDs

device (1 × 1 cm^2^)	*R*_s_ (Ω cm^2^)	*R*_ct_ (Ω cm^2^)	χ^2^	σ (mS cm^–1^)
LEP/BzV-Fc ECD	15.62 ± 0.86	44.76 ± 0.75	1.66 × 10^–4^	13.20 ± 0.17
PVDF-HFP/IL/BzV-Fc ECD	25.20 ± 0.93	92.80 ± 0.99	2.27 × 10^–4^	7.49 ± 0.90
NR/PVDF-HFP/IL/BzV-Fc ECD	18.35 ± 0.26	73.19 ± 0.69	1.38 × 10^–4^	10.81 ± 0.41
QD/PVDF-HFP/IL/BzV-Fc ECD	18.17 ± 0.77	70.99 ± 0.54	1.19 × 10^–4^	11.23 ± 0.63
QD-NR/PVDF-HFP/IL/BzV-Fc ECD	16.75 ± 0.30	48.79 ± 0.21	1.76 × 10^–4^	12.27 ± 0.32

Since the fabricated ECDs encompass a symmetric cell configuration, ionic conductivity (σ) calculations were also carried out to understand the ion transportation behavior. The conductivity values (listed in Table 1) were calculated through the expression^63^ σ = *L*/(*A*·*R*_s_), where *L* is the thickness of the sandwiched GPE membrane and *A* is the electrolyte’s active surface area (1 × 1 cm^2^). The cell constant (*L*/*A*) was obtained by utilizing a commercial electrolyte of known conductivity (NaCl, σ = 12.9 mS cm^–1^) between the sandwiched structure of a dummy ECD cell. The σ values listed in Table 1 show the superiority of filler-included GPEs over filler-less GPEs; the filler-less PVDF-HFP/IL/BzV-Fc ECD displayed the lowest σ (∼7.5 mS cm^–1^), which gradually increased for NR/PVDF-HFP/IL/BzV-Fc ECD (∼10.8 mS cm^–1^) and QD/PVDF-HFP/IL/BzV-Fc ECD (∼11.2 mS cm^–1^). Importantly, QD-NR/PVDF-HFP/IL/BzV-Fc ECD marked a flagship σ value of ∼12.3 mS cm^–1^, which is in the realm of solution-based electrolytes, thus greatly nullifying the low ionic conductivity barrier encountered in GPEs. The enhancement in ionic conductivity is brought by improved EU and VP (Table S3) as well as through the filler–ionic liquid-electrolyte interaction, causing the release of more TBA^+^ and BMIM^+^ cations, thus forming a highly conductive pathway in the GPE matrix.^47,57^ It is noteworthy to mention that BMIMBF_4_ is usually prone to moisture uptake, which might deliver overestimated values of ionic conductivity in the utilized GPE. To avoid such possibilities, BMIMBF_4_ and electrospun polymer electrolytes were long-dried in a vacuum before use. Also, the prepared ECDs were carefully edge-encapsulated to avoid moisture uptake, as mentioned in experimental Section 2.4.

#### 3.6.2 Cyclic Voltammetry

The cyclic voltammetry (CV) analysis of the engineered ECDs, with and without a filler, was performed to investigate the electrochemical kinetics and redox behavior of the utilized electrochromic species in the GPE system. The characteristic redox behavior of the electrochromic species utilized in ECDs is provided in the Supporting Information of this work (Methods S1.2, Figure S3). The CV curves displayed in Figure 5c were recorded at a scan rate and a potential range (maximum) of 100 mV/s and 0–1.25 V (vs Fc), respectively. The EC material (BzV^2+^) and counterion-storage species (Fc) inside the ECD configuration are initially colorless and march toward reduced (BzV^+•^) and oxidized (Fc^+^) states at an externally applied bias, thus displaying a blue color solely arising from BzV^+•^ (as Fc^+^ is colorless). The redox peaks for the Fc/Fc^+^ system could not be observed in Figure 5c due to the peak overlapping with BzV^2+^/BzV^+•^. Figure 5c displays that LEP/BzV-Fc ECD reaches the peak current density (*I*_c_) of ∼2.2 mA/cm^2^ at ∼1.1 V, which is an indication of the maximum attainable reduction (up to the first redox peak) for the EC species utilized in this device. Further, moving from LEP/BzV-Fc ECD to PVDF-HFP/IL/BzV-Fc ECD resulted in a drop in the peak current density (∼1.8 mA/cm^2^) although the reduction potential remained at ∼1.1 V. The remarkable benefits of filler incorporation in the GPE system were evidenced by the lowered redox potential window (∼1.0 V) of NR/PVDF-HFP/IL/BzV-Fc ECD, QD/PVDF-HFP/IL/BzV-Fc ECD, and QD-NR/PVDF-HFP/IL/BzV-Fc ECD, suggesting the higher kinetic ability of the BzV^2+^ ⇌ BzV^+•^ reaction. Additionally, QD-NR/PVDF-HFP/IL/BzV-Fc ECD witnessed a considerable improvement in the peak current density (∼2.1 mA/cm^2^), indicating its better charge carrier ability as compared to the counterpart filler-less GPE-based ECDs. The electrochemical enhancement realized through CV plots in filler-incorporated ECDs is supposed to arise from Lewis acid–base sites provided by ceria nanofillers, which accelerate the redox kinetics by lowering the activation energy barrier.^64^ For representation, the electrochromic process recorded for QD-NR/PVDF-HFP/IL/BzV-Fc ECD during CV testing is displayed in a separately provided Electronic Video File.

Since a reversible electrochemical system follows the Nernst equation (or its derived equations), the current and the scan rate are proportional: *i* ∝ √*v*. This feature was utilized to calculate diffusion coefficient values through the Randles–Sevick equation^65^ for further understanding the mass transfer phenomena in the ECD configuration.
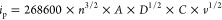
5

where *i*_p_ is the peak current (*A*), *n* is the number of electrons involved in the redox reaction (here 1), *F* is Faraday’s constant (96485.3 C mol^–1^), *A* is the electrode’s active surface area (1 cm^2^), *v* is the scan rate (0.005–0.5 V s^–1^), *C* is the bulk concentration of the electrochromic redox species (5 × 10^–5^ mol cm^–3^), and *D* is the diffusion coefficient (cm^2^ s^–1^) of the reduced electrochromic species, all at 298.15 K. In order to calculate diffusion coefficient values, the ECDs were driven to a range of scan rates ranging from 5 to 500 mV s^–1^, and the obtained CV plots are shown in Figure S4. The *D* values calculated through eq 5 for the LEP/BzV-Fc ECD, PVDF-HFP/IL/BzV-Fc ECD, NR/PVDF-HFP/IL/BzV-Fc ECD, QD/PVDF-HFP/IL/BzV-Fc ECD, and QD-NR/PVDF-HFP/IL/BzV-Fc ECD were found to be 5.77 × 10^–7^, 2.39 × 10^–7^, 4.75 × 10^–7^, 3.27 × 10^–7^, and 4.72 × 10^–7^ cm^2^/s, respectively. The obtained *D* values aligned well with the *R*_s_ values listed in Table 1, indicating the sluggish diffusion due to the higher impedance of the bulk electrolyte in PVDF-HFP/IL/BzV-Fc ECD, which gradually decreased upon filler incorporation, as seen in the advanced diffusion coefficient values. One important aspect remains the possibility of the piezoelectric effect due to the presence of a typical piezoelectric material, PVDF-HFP, in our fabricated ECDs. The visuals of the experimental setup designed for the probing of the piezoelectric effect are displayed in Figure 5d–f. The corresponding text discussion is presented in Section S1.3 of the Supporting Information. As probed, no meaningful evidence of piezoelectricity was observed in the tested ECD.

#### 3.6.3 Absorbance and Transmittance Analyses

**Figure 6:**
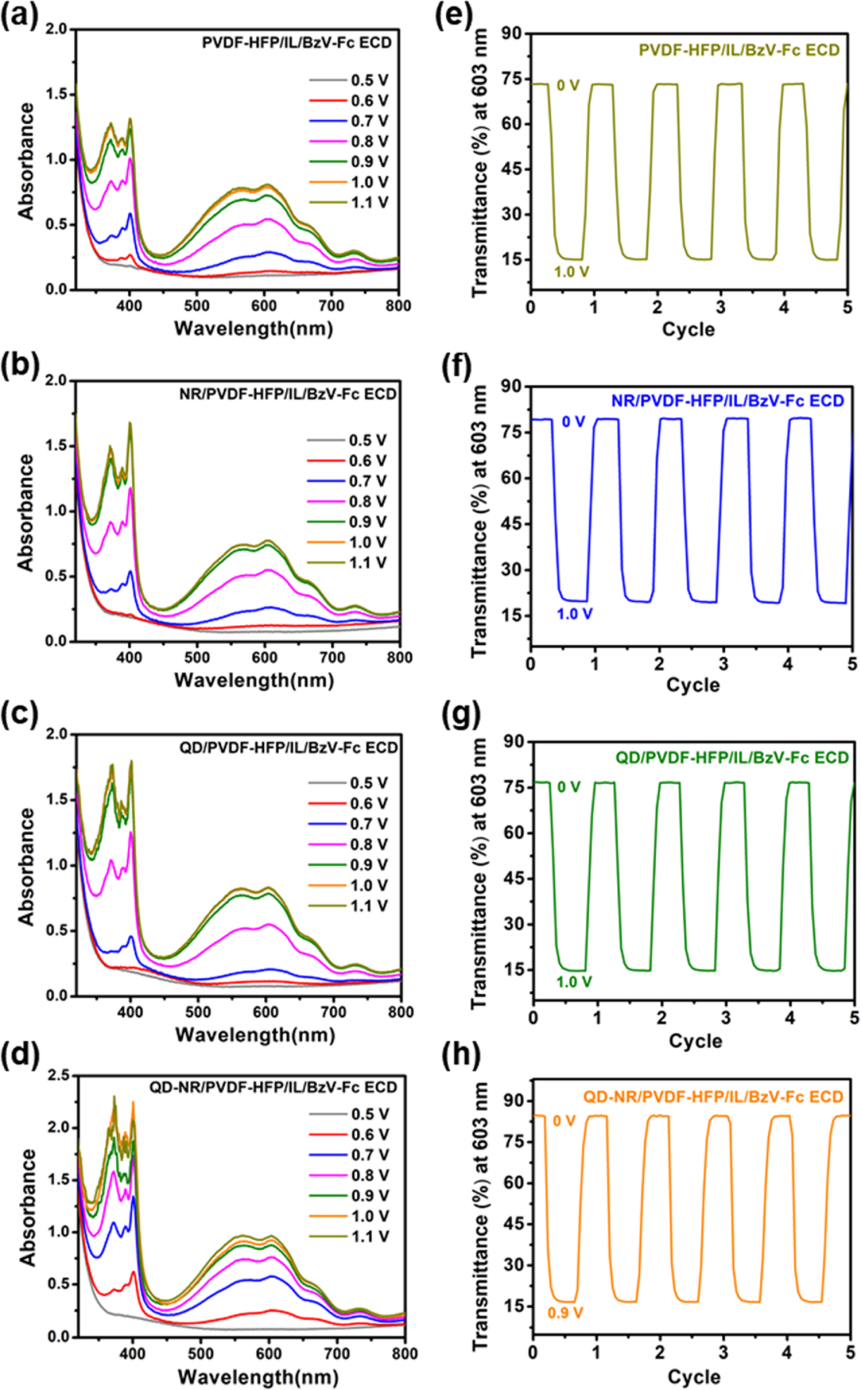
Absorbance and transmittance spectra of (a, e) PVDF-HFP/IL/BzV-Fc ECD, (b, f) NR/PVDF-HFP/IL/BzV-Fc ECD, (c, g) QD/PVDF-HFP/IL/BzV-Fc ECD, and (d, h) QD-NR/PVDF-HFP/IL/BzV-Fc ECD. The absorbance spectra were recorded in the UV–vis range by switching the ECDs from 0 to 1.1 V through a step voltage increment of 0.1 V. The transmittance spectra were obtained by switching the ECDs between bleached (0 V) and colored states (0.9/1.0 V), retaining 10 s in each stage at λ_max_ = 603 nm.

**Figure 7:**
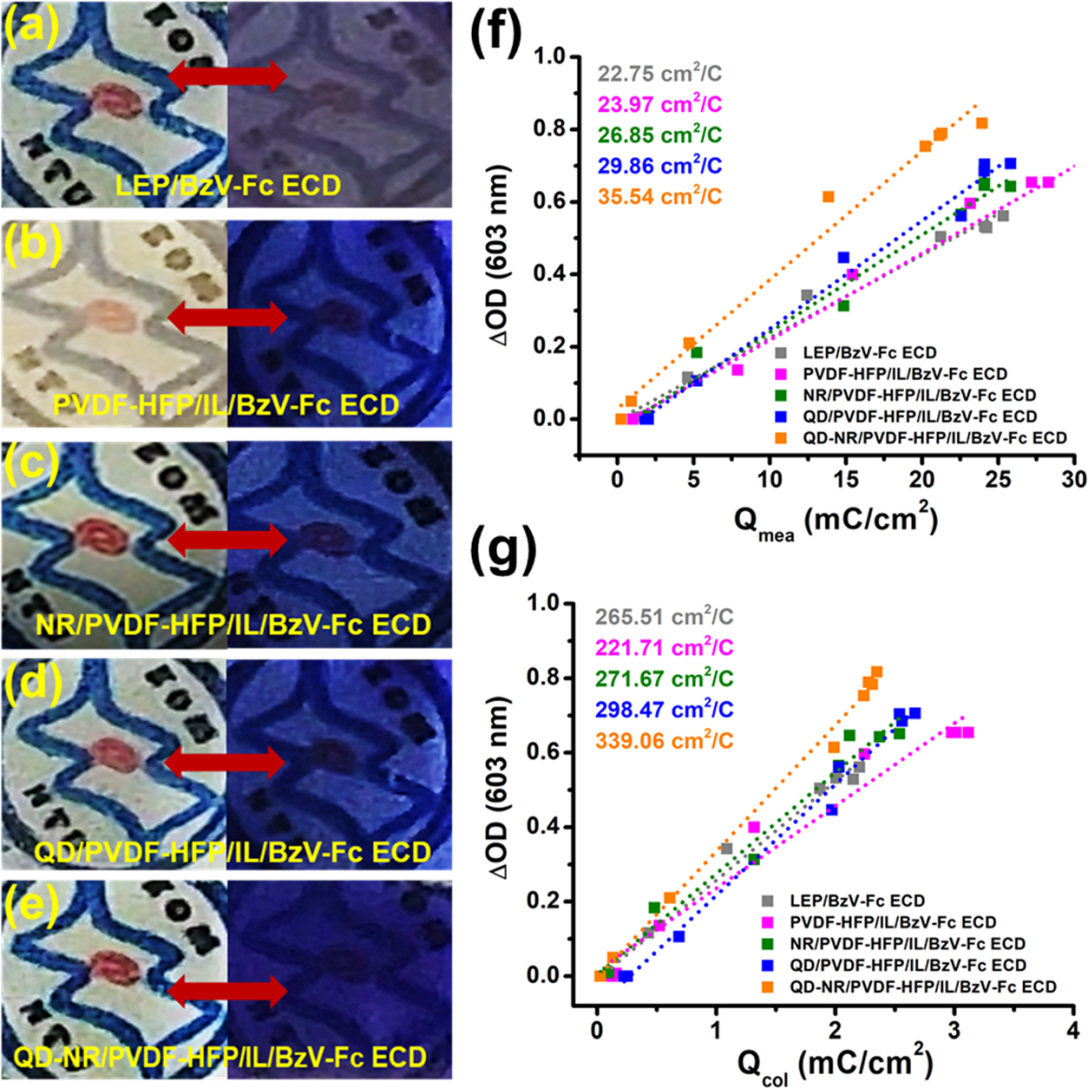
Bleached and colored state images of the representative (a) LEP/BzV-Fc ECD, (b) PVDF-HFP/IL/BzV-Fc ECD, (c) NR/PVDF-HFP/IL/BzV-Fc ECD, (d) QD/PVDF-HFP/IL/BzV-Fc ECD, and (e) QD-NR/PVDF-HFP/IL/BzV-Fc ECD. (f) Conventional and (g) effective coloration efficiency plots of prototype, filler-less, and CeO_2_ filler-incorporated GPE-based ECDs.

Parameters such as high optical density/absorbance in a colored state and good optical tunability with the least external voltage requirement are the desired features for the candidacy of an excellent ECD. For absorbance analysis in our study, the ECDs were driven from 0 to 1.1 V by a step voltage increment of 0.1 V in the UV–vis region (300–800 nm), and the obtained spectra are shown in Figures 6a–d and S5a. It is apparent from Figure S5a that BzV has two characteristic peaks at ∼400 and ∼603 nm, at which the change in optical density (ΔOD) attains maximum values. Further, careful observation of the absorbance spectra in Figure 6a–d suggested that valuable attainment in ΔOD was realized only up to ∼1.0 V, and any further stepping of voltage caused a trivial enhancement in the optical density. This phenomenon is expected as the optical traits delivered by BzV, up to ∼1.0 V, majorly belong to the first redox pair (Figure 5c). Further enhancement in ΔOD will only be realized if the induced potential triggers the second reduction reaction, which appears to emerge beyond ∼1.2 V. As apparent from Figures 6a–c and S5a, the ΔODs (603 nm) for LEP/BzV-Fc ECD, PVDF-HFP/IL/BzV-Fc ECD, NR/PVDF-HFP/IL/BzV-Fc ECD, and QD/PVDF-HFP/IL/BzV-Fc ECD were found to be ∼0.67, ∼0.75, ∼0.77, and ∼0.82, all obtained at 1.0 V. Interestingly, QD-NR/PVDF-HFP/IL/BzV-Fc ECD soared to an outstanding ΔOD of ∼0.91 with a voltage stimulus of only 0.9 V, as seen in Figure 6d. Due to this remarkable benefit, the QD-NR/PVDF-HFP/IL/BzV-Fc ECD was determined to operate between 0 and 0.9 V, which is 100 mV lower than the otherwise operated voltage window (0–1.0 V) for the utilized ECDs. Additionally, the transmittance change (Δ*T*) ability of the as-mentioned ECDs was probed by shuttling the ECDs between 0 and 1.0 V (10 s stay in each state) at 603 nm (except for QD-NR/PVDF-HFP/IL/BzV-Fc ECD, which was operated in the 0–0.9 V window). The recorded square pulse spectra for dynamic transmittance are displayed in Figure 6e–h, and the corresponding values are quantified in Table 2. The obtained Δ*T* values displayed a similar trend to that of ΔOD values. Concisely, LEP/BzV-Fc ECD exhibited (Figure S5b) the lowest Δ*T* (∼54.7%), which, upon GPE substitution, improved for PVDF-HFP/IL/BzV-Fc ECD (∼57.3%). Further, NR/PVDF-HFP/IL/BzV-Fc ECD and QD/PVDF-HFP/IL/BzV-Fc ECD achieved increased Δ*T* of ∼60.5 and ∼62.3%, respectively, a considerable improvement for filler-based ECDs in comparison to the filler-less counterparts. A remarkable Δ*T* of ∼68.6% was registered for the QD-NR/PVDF-HFP/IL/BzV-Fc ECD in a small potential window of 0–0.9 V, which is a staggering improvement of ∼25.4 and ∼19.7% in Δ*T* compared to solution-type (LEP/BzV-Fc ECD) and filler-less (PVDF-HFP/IL/BzV-Fc ECD) equivalent, respectively, of course with a desirable energy-saving feature (∼100 mV reduction in operating voltage). For the physical realization of optical enhancement in bleached and colored states, the real-time images of engineered ECDs are presented in Figure 7a–e. The noteworthy increment in absorbance and optical contrast for filler-based ECD is assisted by proliferated Ce^4+^ ↔ Ce^3+^ redox centers (Table S1), providing abundant interaction sites for the BzV^2+^⇌ BzV^+•^ redox reaction, which in turn causes a rich color and bleached states.

**Table 2:** Response Time, Transmittance in the Bleached and Colored State, and Transmittance Change Values of Utilized ECDs at 603 nm

device	τ_c_ (s)	τ_b_ (s)	*T*_b_/*T*_c_	Δ*T* (%)
LEP/BzV-Fc ECD	2.1	2.4	80.1/25.4	54.7
PVDF-HFP/IL/BzV-Fc ECD	3.1	2.8	72.8/15.5	57.3
NR/PVDF-HFP/IL/BzV-Fc ECD	2.8	2.3	79.8/19.3	60.5
QD/PVDF-HFP/IL/BzV-Fc ECD	2.7	2.1	77.2/14.9	62.3
QD-NR/PVDF-HFP/IL/BzV-Fc ECD	1.8	1.9	84.9/16.3	68.6

#### 3.6.4 Response Time and Coloration Efficiency Analysis

As an important benchmark, the response time or switching rapidity of ECDs from the bleached state to the colored state and vice versa was determined in our study. The coloration time (τ_c_) and the bleaching time (τ_b_) were ascertained as the time required to reach 95% of the maximum attainable Δ*T* on either side of the ECD switching. The τ_c_ and τ_b_ values extracted from the square pulses of the transmittance spectra (Figures 6e–h and S5b) are mentioned in Table 2. The values listed in Table 2 suggest that the fast diffusion mechanism in LEP/BzV-Fc ECD resulted in short τ_c_ and τ_b_ (∼2.1 and ∼2.4 s), which was prolonged in PVDF-HFP/IL/BzV-Fc ECD due to sluggish diffusion kinetics (∼3.1 and ∼2.8 s). The incorporation of nanofillers in the ECD markedly addressed the response time delay issue and resulted in improved τ_c_ and τ_b_ for NR/PVDF-HFP/IL/BzV-Fc ECD (∼2.8 and ∼2.3 s), QD/PVDF-HFP/IL/BzV-Fc ECD (∼2.7 and ∼2.1 s), and QD-NR/PVDF-HFP/IL/BzV-Fc ECD (∼1.8 and ∼1.9 s) as displayed in Table 2. The improvement in response time could be well corroborated with enhanced diffusion coefficient values realized for filler-based ECDs, as discussed in Section 3.6.2.

The coloration efficiency (CE) analysis for the representative ECDs was also carried out, as it is the bridging factor between electrochemical and optical performance. Calculation-wise, CE (η) = ΔOD/*Q*_mea_, where *Q*_mea_ refers to the measured charge density and can be evaluated by integrating amperometric time–current signals obtained at different applied potentials (0–1.1 V). An efficient ECD is expected to show higher ΔOD at low charge injection for a higher coloration efficiency. The CE of the as-prepared ECDs was calculated through the slope of the ΔOD vs *Q*_mea_ plot, as shown in Figure 7f. Usually, different steady-state current densities are observed in amperometric time–current signals, which leads to deceptive measured charge densities and, therefore, raises concerns about CE in ECDs. Our previously introduced concept of effective coloration efficiency (η_e_)^66^ addresses this issue by removing interference from the steady-state current. The effective CE thus can be represented as η_e_ = ΔOD/*Q*_col_, where *Q*_col_ refers to effective charge density. The effective CE of the as-prepared ECDs was realized through the ΔOD vs *Q*_col_ plot, as highlighted in Figure 7g.

The conventionally calculated η of the as-prepared ECDs showed an incremental pattern from solution-based LEP/BzV-Fc ECD (∼22.75 cm^2^/C) to filler-less PVDF-HFP/IL/BzV-Fc ECD (∼23.97 cm^2^/C) and filler-based NR/PVDF-HFP/IL/BzV-Fc ECD (∼26.85 cm^2^/C), QD/PVDF-HFP/IL/BzV-Fc ECD (∼29.86 cm^2^/C), and QD-NR/PVDF-HFP/IL/BzV-Fc ECD (∼35.54 cm^2^/C). Unlike η, the calculation of η_e_, as shown in Figure 7g, suggested a better CE performance for LEP/BzV-Fc ECD (∼265.51 cm^2^/C), followed by a substantial drop when transitioned to GPE-based PVDF-HFP/IL/BzV-Fc ECD (∼221.71 cm^2^/C). This drop in CE is reasonable in view of the higher *R*_s_ and *R*_ct_ impedance prevailing in filler-less GPE-based ECD, as seen in Table 1. Further, the filler-based NR/PVDF-HFP/IL/BzV-Fc ECD and QD/PVDF-HFP/IL/BzV-Fc ECD demonstrated modest (∼271.67 cm^2^/C) to good (∼298.47 cm^2^/C) enhancement in η_e_ as compared to LEP/BzV-Fc ECD. The QD-NR/PVDF-HFP/IL/BzV-Fc ECD demonstrated a flagship CE of ∼339.06 cm^2^/C, far exceeding the LEP/BzV-Fc ECD and its other counterparts. The enhancement in η and η_e_ for filler-hosted ECDs could be understood from the higher Δ*T* achieved at a relatively low voltage input. In other words, high transmittance change achieved at a low operating voltage (and hence minimal charge injection) results in greater CE values, as observed for QD-NR/PVDF-HFP/IL/BzV-Fc ECD.

#### 3.6.5 Stability Analysis

**Figure 8:**
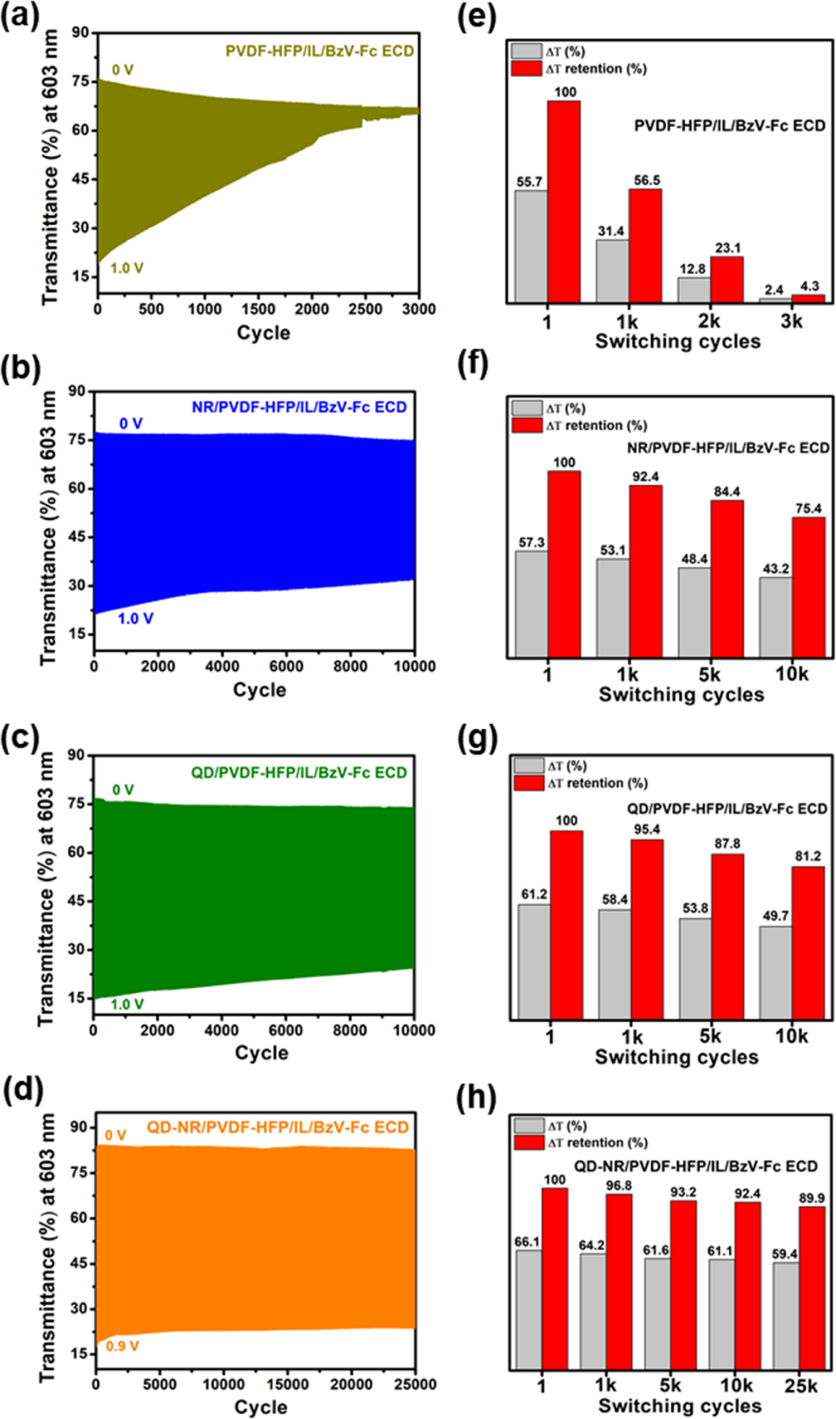
Cycling stability plots with quantified optical contrast (Δ*T*) and Δ*T*-retention for (a, e) PVDF-HFP/IL/BzV-Fc ECD, (b, f) NR/PVDF-HFP/IL/BzV-Fc ECD, (c, g) QD/PVDF-HFP/IL/BzV-Fc ECD, and (d, h) QD-NR/PVDF-HFP/IL/BzV-Fc ECD at λ_max_ = 603 nm. The utilized ECDs were switched between the bleached state (0 V) and colored state (0.9/1.0 V), holding 5 s in each state.

The switching stability or long-term stability is the most critical characteristic of GPE-based ECDs, which has hindered their commercial applicability for years. Although the GPE’s inclusion in ECD embraces state-of-the-art trends, the practical scalability of GPE-based ECDs is still hindered by their poor long-term cycling stability. More importantly, while parameters such as transmittance change, switching time, and coloration efficiency are easily available in published reports, the long-term cycling study of GPE-based ECDs is rare. To address this ambiguity, the as-fabricated ECDs were tested for long-term stability by switching them between bleached (0 V) and colored (0.9/1.0 V) states—retaining 5 s in each state. The obtained plots for cycling stability are highlighted in Figures 8a–d and S5c. The Δ*T* values and a sister parameter known as Δ*T*-retention are calculated and quantified as shown in Figures 8e–h and S5d. The Δ*T*-retention after *n*-cycles indicates the ECD’s ability to retain its initial optical contrast (Δ*T*_int_). It is usually agreed that viologen-based ECDs are highly stable in liquid-phase electrolytes, and this trait can be confirmed by the high Δ*T*-retention of LEP/BzV-Fc ECD (Figure S5c) even after 10k cycles: Δ*T*_10K_ ∼ 93% Δ*T*_int_. In contrast, the transition from a stable solution type to GPE witnessed a continuous depletion in the switching stability of PVDF-HFP/IL/BzV-Fc ECD. The device could hardly endure effective switching beyond 1k cycles as apparent in Figure 8a,e: Δ*T*_1K_ ∼ 56% Δ*T*_int_. The drastic fall in Δ*T* was rooted in the draining colored state of PVDF-HFP/IL/BzV-Fc ECD, which suggests that reduction of the viologen dication (BzV^2+^⇌ BzV^+•^) at the electrode surface is greatly hindered. The reasons are primarily attributed to (i) prevailing high series and charge transfer resistance (Table 1) inducing sluggish diffusion and high barrier for the BzV^2+^⇌ BzV^+•^ redox reaction and (ii) electrolyte’s structural deformity in filler-less GPEs due to lack of mechanical strength (Figure 4b). The incorporation of nanofillers in the GPE matrix witnessed astounding stability benefits, as can be seen from Figure 8b–d,f–h; the NR/PVDF-HFP/IL/BzV-Fc ECD and QD/PVDF-HFP/IL/BzV-Fc ECD retained ∼75 and ∼81% Δ*T*_int_ after 10k switching cycles, which is a landmark development in cycling stability compared to its filler-less counterpart. Interestingly, QD-NR/PVDF-HFP/IL/BzV-Fc ECD showed ∼92% Δ*T*_int_ after 10k switching cycles, which encouraged us to check the device stability further, and the obtained results were notable; the device maintained ∼90% Δ*T*_int_ after 25k switching cycles. To our delight, QD-NR/PVDF-HFP/IL/BzV-Fc surpassed the cycling stability behavior of the solution-type LEP/BzV-Fc ECD, as displayed in Figures 8d,h and S5c,d. The obtained findings are the rare demonstration where a GPE version of viologen-based ECD outclassed its highly stable solution-type counterpart on the grounds of cycling stability. For comparative insights, some recent studies announcing major developments in gel/solid polymer-based ECDs have been indexed alongside our findings in Table S5. The underlying effect brought by CeO_2_ nanofillers was stepwise understood from the series of cycling stability tests, as detailed in Section S1.4. The possibilities of heat generation during the ECD switching were also investigated thoroughly, as discussed in Section S1.5 of the Supporting Information. Considering that CeO_2_ demonstrates photocatalytic activity, the filler-based ECDs were also tested in an irradiated atmosphere (100 mW cm^–2^, AM 1.5G) to probe the possible degradation effect. The setup and CV plots obtained for the irradiated filler-based ECDs are highlighted in Figure S8a–d. A slight drop in the current density was seen for the NR and QD-based ECDs after 500 CV cycles, as shown in Figure S8b,c, respectively. However, no such severe degradation was noticed. Moreover, the QD-NR/PVDF-HFP/IL/BzV-Fc ECD retained ∼100% current density after 500 cycles, as apparent from Figure S8d. The obtained results suggested that the CeO_2_ filler-based ECDs can exhibit good endurance against solar irradiation.

Since the QD-NR-based electrolyte delivered the flagship performance, its suitability with flexible substrates (ITO/PET) was also examined through a flexible ECD assembly (labeled as QD-NR/PVDF-HFP/IL/BzV-Fc FECD). The visuals of the fabricated FECDs are shown in Figure S9a–c. The QD-NR/PVDF-HFP/IL/BzV-Fc FECD in its as-prepared state showed remarkably stable behavior when switched for the 1000 cycles, as can be seen in Figure S9d. However, to investigate the mechanical endurance of the prepared electrolyte, the FECDs were manually bent for *n* (10, 50, and 100) times before switching. It can be noticed from Figure S9e,f that the high reversibility of QD-NR/PVDF-HFP/IL/BzV-Fc FECD was well maintained up to 50 bends, with almost 90% retention of Δ*T*_int_. However, a slight drop in overall Δ*T*_int_ was observed after bending as compared to the as-prepared FECD. The Δ*T*_int_ of QD-NR/PVDF-HFP/IL/BzV-Fc FECD did not drop (only ∼20%) severely even after 100 bends, as depicted in Figure S9g. Overall, the QD-NR filler-based electrolyte exhibited good endurance in robust environments, hence signaling its candidacy for high-performance FECDs.

#### 3.6.6 Haze Analysis

**Figure 9:**
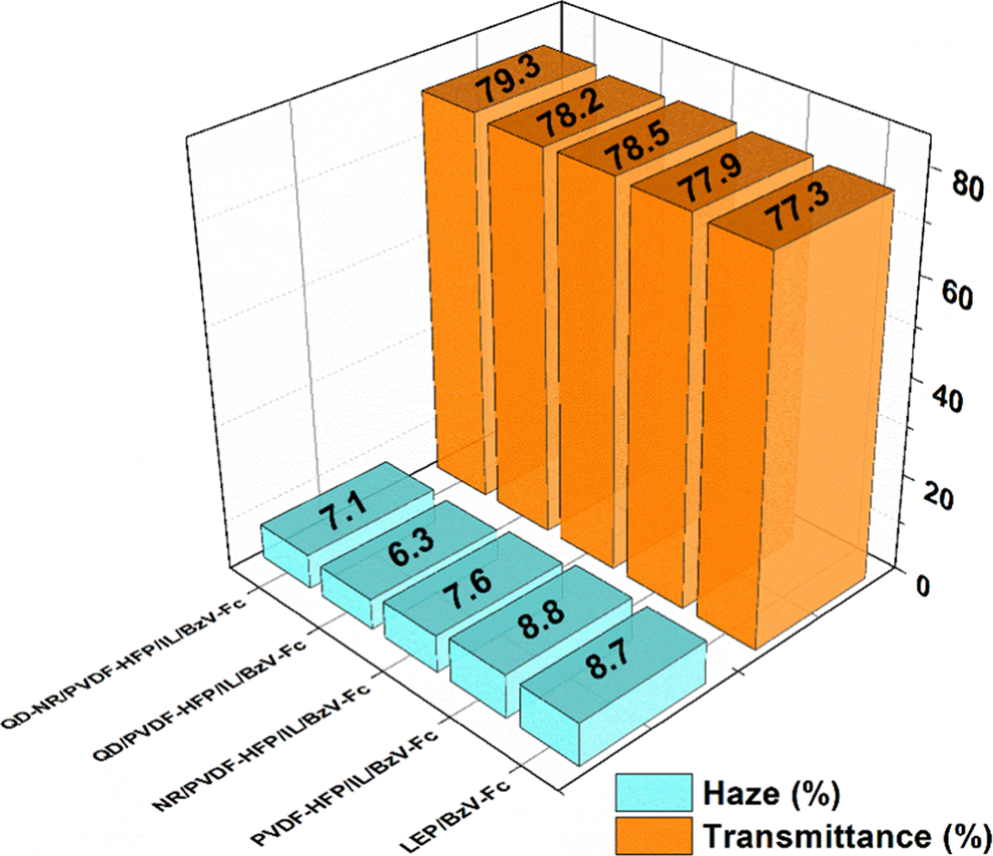
Haze and corresponding transmittance values of the ECDs in their neutral state.

Being a vital index of the device’s visibility, the haze of the ECDs was recorded with corresponding transmittance values, as shown in Figure 9. It was noticed that all of the utilized ECDs maintained the desired low haze (<10%) and high transmittance values (>75%) in their neutral state. It was also observed that the filler-based ECDs showcased slightly lower haze (∼7.6, ∼6.3, and ∼7.1) values compared to filler-less ECDs (∼8.7 and ∼8.8). This might be due to the complementing refractive indices developed within the electrolyte matrix owing to distinct benefits endowed by nanofillers, as seen earlier in Sections 3.4 and 3.5. The reasons can also be associated with the relatively smaller sizes (QD ∼ 7 nm; NR ∼ 55 nm) of the utilized nanofillers, thus greatly nullifying the possibility of Rayleigh scattering, which otherwise might have led to high haze values. Overall, no adverse effect of nanofiller incorporation was registered in the ECDs; instead, an improvement in haze (low) was seen due to its size-specific utilization.

#### 3.6.7 Proposed Mechanism for the Underlying Effect of CeO_2_ Nanofillers

**Figure 10:**
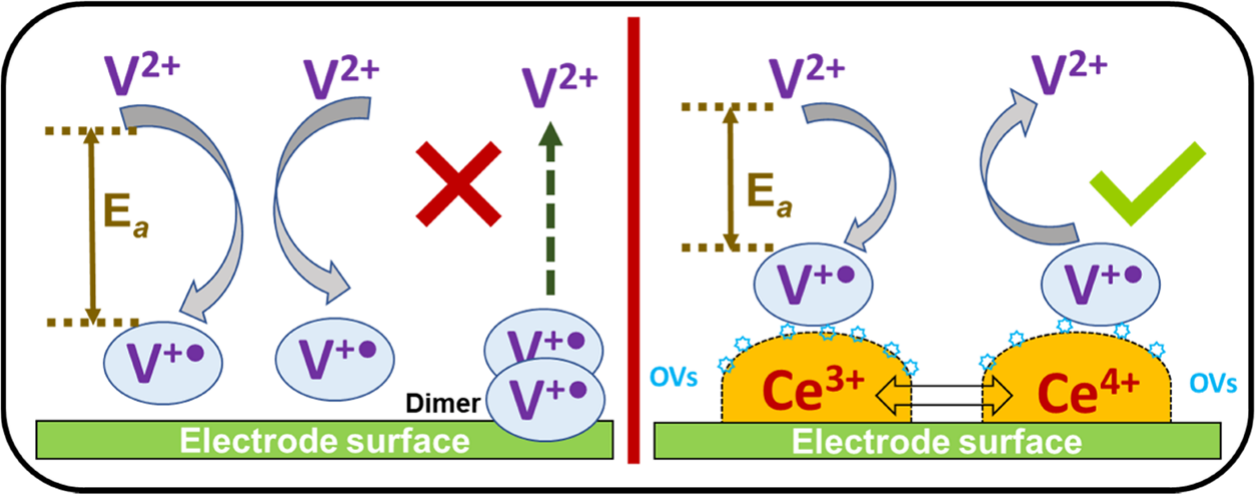
Proposed mechanism highlighting the effects induced by CeO_2_ nanofillers.

The proposed mechanism for the enhancement brought by CeO_2_ nanofillers is depicted in Figure 10. The filler-less electrode surface is proposed to be readily available for dimerization reaction due to the high affinity of viologen radicals (V^+•^) to form dimers in its unstable state. Unlike the viologen radical cation, the formed dimers (2 V^+•^) are irreversible in nature, thus greatly impeding the back-oxidation reaction responsible for the ECD’s bleaching (Figure 10, left half). In the long term, the continuous formations of these dimers cause high charge transfer resistance on the electrode surface, thus greatly diminishing the device’s cycling stability. This behavior can be well confirmed by the poor cyclic stability of filler-less ECD, as highlighted in Figure 8a. In contrast, the presence of nanofillers in the ECD matrix delivers numerous Lewis acid–base locations along with rich oxygen vacancies (OVs, Ce^3+^) near the electrode region, which serves as an adsorption site for viologen radical cations (V^+•^), as shown in Figure 10 (right half). The temporary stabilization of the viologen radical (V^+•^) through created adsorption sites suppresses the detrimental dimer formation on the electrode surface, thus maintaining the required low charge transfer resistance during the switching. Although the nanorod and quantum dot architecture of CeO_2_ provides numerous interaction sites due to the high reactivity of the prevailing (100) and (110) planes, their interaction with the adsorbed viologen radical is proposed to be relatively weak, thus still favoring the detrimental dimerization event to some extent. In contrast, the rugged junctions created at the QD/NR interface render abundant reactive (311) planes in the QD-NR architect, in addition to the distributed (100) and (110) planes, as earlier confirmed through TEM analysis. This added proliferation of interactive planes (and thus OVs) in the QD-NR structure allows relatively strong interactions with the viologen radical, thus mitigating the dimer formation more effectively. The explanation presented here can be well associated with the cycling stability behavior of CeO_2_ filler-based ECDs, where QD-NR-based ECDs demonstrated enhanced stability compared to their individual NR and QD-based counterparts, as seen in Figure 8b–d. In addition to the mechanism presented above, the possibilities of additional benefits are also perceived in the form of meticulous Ce^3+^/Ce^4+^ shuttling, which parallelly smoothens the reaction kinetics of V^+•^/V^2+^. Various reports have confirmed that the enormous Lewis acid–base pair available^42,64^ on the CeO_2_ surface greatly reduces the activation barrier for reactants. This feature can be well associated with the enhanced electrochemical performance of QD-NR-based ECD, which attained full reversibility in the minute voltage window of only ∼0.9 V, hence delivering a high optical contrast.

## 4 Conclusions

To summarize, we developed a novel class of gel-polymer electrolytes incorporating CeO_2_ nanorods (NR), quantum dots (QDs), and their hybrid nanostructure (QD-NR) as a nanofiller to yield astounding performance in viologen-based ECDs. In a scarce event, a GPE-based ECD outperformed its solution-type counterpart on a yardstick of critical ECD parameters such as operating voltage window, optical tunability, response time, and cycling endurance (shown in Table 3). The QD-NR/PVDF-HFP/IL/BzV-Fc ECD marked a high Δ*T* of ∼68.6% at a remarkably low voltage window of 0–0.9 V, thus giving rise to a landmark development in the family of high voltage-driven gel/solid electrolyte-based ECDs. The benefit of CeO_2_ fillers also led to considerable improvement in electrochromic properties as NR/PVDF-HFP/IL/BzV-Fc ECD, QD/PVDF-HFP/IL/BzV-Fc ECD, and QD-NR/PVDF-HFP/IL/BzV-Fc ECD marked high coloration efficiencies of 271.67, 298.47, and 339.06 cm^2^/C at 603 nm. The outstanding cycling stability (∼90% retention of Δ*T* after 25,000 switching cycles) witnessed for QD-NR/PVDF-HFP/IL/BzV-Fc ECD offers a promising route toward the yet-to-realize commercialization of GPE-based ECDs. The astounding results showcased by ECDs stemmed from ceria nanofillers, which proliferated abundant redox centers and enhanced oxygen vacancies in the GPE matrix. We believe this study not only marks a landmark development in the field but also carries the potential to attract researchers’ interest in the marvels of filler-based ECDs.

**Table 3:** Comparison of QD-NR Doped GPE-Based ECD with Its Solution-Type Counterpart

ECD	λ_max_ (nm)	opr. window (V)	Δ*T* (%)	τ_c_/τ_b_ (s)	η_e_ (cm^2^/C)	Δ*T*-retention (%) after 25,000 cycles
LEP/BzV-Fc ECD	603	1.0	54.7	2.1/2.4	265.5	87.6
QD-NR/PVDF-HFP/IL/BzV-Fc ECD	603	0.9	68.6	1.8/1.9	339.1	89.9

## Supporting Information Available

Methods, estimation of redox centers, optical contrast of ECDs, determination of electrolyte uptake and volumetric porosity, transmittance and stability analysis, TEM analysis, cyclic voltammetry plots, UV–vis absorbance spectra, thermal imaging (IR) analysis, photostability analysis, stability plots of flexible ECDs (PDF)
Electrochromic process recorded for QD-NR/PVDF-HFP/IL/BzV-Fc ECD during the CV testing (MP4)
