# Supplementary material for: Demonstration of a Gel-Polymer Electrolyte-Based Electrochromic Device Outperforming Its Solution-Type Counterpart in All Merits: Architectural Benefits of CeO2 Quantum Dot and Nanorods
Source: ACS Appl Mater Interfaces. 2024 Jan 19;16(4):4958–74. doi: 10.1021/acsami.3c16506 (PMC10835657; doi:10.1021/acsami.3c16506)
Supplement: Supplementary file 1 — am3c16506_si_001.pdf [file am3c16506_si_001.pdf]

Supporting Information

Demonstration of a Gel Polymer Electrolyte-Based Electrochromic Device Outperforming Its Solution Type Counterpart in All Merits: Architectural Benefits of CeO_2 Quantum Dot and Nanorods

Gaurav Kumar Silori, [†] Subashchandrabose Thoka, [†] Kuo-Chuan Ho [†,‡,§*]

[†] *Department of Chemical Engineering, National Taiwan University, Taipei 10617, Taiwan*

[‡] *Institute of Polymer Science and Engineering, National Taiwan University, Taipei 10617, Taiwan*

[§] *Advanced Research Center for Green Materials Science and Technology, National Taiwan University, Taipei 10617, Taiwan*

[*]*Corresponding author: Tel.: +886–2–2366–0739; Fax: +886–2–2362–3040*

E–mail: kcho@ntu.edu.tw

S1. Methods

S1.1 Determination of CeO_2 loading for optimal ECD performance

The optimized filler loading in this work was determined through the utilization of filler-incorporated GPE in ECDs with different loading amounts. The CeO_2 nanorods were chosen for utilization in four different concentrations of 0.5, 1.0, 1.4, and 2.0 wt % (PVDF-HFP basis). The average aspect ratio of synthesized NR nanofillers was determined to be 55:5, as can be seen from highlighted TEM image in **Fig. S1**. For performance analysis of ECD with different filler-loading, a vital parameter of ECD known as transmittance change (ΔT) or optical contrast was taken into consideration. The obtained transmittance spectra (at 603 nm) for ECDs with different filler loading are shown in **Fig. S2,** and the corresponding ΔT values are presented in **Table S2.** The ΔT value for filler-less PVDF-HFP/IL/BzV-Fc ECD listed in **Table S2** for comparison purposes is called from **Table 2** of the main text**.** It was realized that low-filler loading (0.5 wt%) did not contribute to ΔT of $NR_{0.5}$/PVDF-HFP/IL/BzV-Fc ECD as compared to filler-less PVDF-HFP/IL/BzV-Fc ECD as seen in **Table S2**. However, the benefits were realized in increased filler-loading (1 wt%), which displayed an ΔT of ~60% in $NR_{1.0}$/PVDF-HFP/IL/BzV-Fc ECD. Further increment in filler-loading demonstrated an ΔT of ~58.7% and ~53.1 % for $NR_{1.5}$/PVDF-HFP/IL/BzV-Fc ECD and $NR_{2.0}$/PVDF-HFP/IL/BzV-Fc ECD, respectively, which is an inferior performance compared to $NR_{1.0}$/PVDF-HFP/IL/BzV-Fc ECD. The optimized performance for 1wt % filler loading lies in the fact that lower filler loading doesn't trigger the required enhancement in GPEs, while the higher loading of filler causes transmittance drop due to light scattering stemming from undesired agglomeration/bed formation in the GPE system.

S1.2 Characteristic redox behavior of electrochromic species utilized in ECDs

The three-electrode system was employed to realize the intrinsic characteristics of utilized viologen species [BzV:1,1′-bis(4-fluorobenzyl)-4,4′-bipyridine-1,1′-diium tetrafluoroborate]. Briefly,

0.01 M viologen species was dissolved in PC solution using 0.1 M $TBABF_4$ as a supporting electrolyte, and the mixture was placed in a cell cuvette. Further, an ITO with an active area of 1.0 × 1.0 cm^2 was clamped inside the cell to work as a working electrode, while Pt and a homemade Ag/Ag^+ were used as counter and reference electrodes, respectively. The cyclic voltammetry at a scan rate of 100 mV/s was performed on an as-prepared three-electrode system using a negative potential bias as viologens are cathodically coloring materials displaying blue color in the reduced state. The obtained CV plot is displayed in **Fig. S3** with two redox peaks appearing in the forward cycling of BzV; the first reduction peaks between -0.5 to -0.9 V showcases the reduction of viologen dictation into radical cation ($BzV^{2+} \rightleftharpoons BzV^{+\bullet}$) while the subsequent second peak between -0.9 and -1.2 V drives unstable radical cation into neutral ion state ($BzV^{+\bullet} \rightleftharpoons BzV^0$). The reverse cycling takes the viologen species back into its second and first oxidation states, respectively, thus completing the redox loop, as evident from **Fig. S3**. More often, the second redox peak in viologens causes irreversibility and could be avoided to realize the better cycling life of electrochromic material.

S1.3 Investigation of the piezoelectric effect in PVDF-HFP-based ECD

The utilized polymer (PVDF-HFP) in our fabricated ECDs is a typical piezoelectric material. Thus, a representative device (QD-NR/PVDF-HFP/IL/BzV-Fc ECD) was investigated for the effect of piezoelectricity, which might possibly originate during any exposed applied stress or heat (speculated to generate from coloration process). First, the effect of stress was probed by simply monitoring the device's open circuit voltage (OCV) before and after the applied stress, as shown in **Fig. 5d,e**. The OCV of tested ECD is expected to alter upon a weight press due to the accumulation of charge observed in the piezoelectric materials. A metal standard (~200 g) was used for employing stress over the ECD’s active area, as shown in **Fig. 5e**. As apparent in **Fig.s 5d,e,** the ECD registered no noticeable change in OCV (only 0.1 mV) upon applied stress, thus discouraging the possibilities of piezoelectricity rendered from the PVDF-HFP. This behavior may be attributed to the presence of a spacer (DuPont 60 µm Surlyn® frame) used during the fabrication of the ECD cell, which significantly nullifies the

effect of applied stress on the gel-polymer electrolyte. Moreover, to examine the probability of piezoelectric effect emerging from continuous coloration/bleaching, the device was subjected to 1000 CV cycles in a voltage range and scan rate of 0 ~ 1.1 V and 100 mV/s, respectively. The OCV was immediately recorded as soon as the 1000th cycle was completed, as displayed in **Fig. 5f**. As compared to the pristine state (**Fig. 5d**), no observable change in OCV was noticed after the 1000 CV cycles of ECD, thus indicating that either the generated heat was too small to trigger any piezoelectric effect or no measurable heat generation occurred in the device during the electrochromic process. This observation was further supported by the IR thermal imaging of the ECD recorded during the bleaching and coloration process, as shown in **Fig. S7a,b.** A temperature increment of only ~1.1 ^{0}C was observed in the colored state of ECD compared to the bleached state, indicating a weak heating effect. To summarize, no meaningful evidence of piezoelectricity was observed in our fabricated ECD due to an inadequate stress or heat effect.

S1.4 Discussion on systematic incorporation of X (=BzV, BzV-Fc, QD-NR/BzV, QD-NR/BzV-Fc) in the host PVDF-HFP/IL ECD

Four ECD configurations were prepared by systematically incorporating X (=BzV, BzV-Fc, QD-NR/BzV, QD-NR/BzV-Fc) to host PVDF-HFP/IL ECD, as to distinctively understand the effect brought by nanofillers. The fabricated ECDs were shuttled between their bleached and colored state by employing a potential bias of 0.0 and 1.0 V, respectively. The obtained ΔT and stability behavior (1000 cycles) of ECDs are highlighted and quantified in **Fig. S6** and **Table S4,** respectively. It was observed that ECD with bare BzV was able to achieve ΔT of ~56% at 603 nm, though degraded abruptly (**Fig. S6e**) from both bleached and colored states indicating the poor reaction kinetics without Fc and nanofillers. In contrast, when the host ECD was inducted with the BzV-Fc combo, a considerable improvement was seen in cycling stability, though only a marginal color enhancement was observed in the bleaching side of the ECD, suggesting that Fc positively contributes to the back-oxidation reaction of $BzV^{+\bullet} \rightleftharpoons BzV^{2+}$. This observation is in good agreement with previous reports [1,2].

Moreover, upon fillers' (here QD-NR) utilization with BzV, a significant enhancement was observed in the bleached state of the host device, as seen in **Fig. S6c** and **Table S4**, though a drop in colored state was also observed. This phenomenon indicated that while the CeO_2 fillers provide enough redox interactive sites towards the cathodic direction (or more negative potential), their functionality still needs support from the counter electrode species (Fc here) at the anodic side. This assumption was validated by further induction of counter electrode species Fc in the filler-BzV duo, which demonstrated excellent coloration improvement at both the cathodic and anodic sides, as seen in **Fig. S6d**. The switching ability of both bare filler and Fc inducted ECDs, showcased a competent beginning however dropped severely in the later cycles, as evidenced by **Fig. S6f,g**. This issue was addressed when Fc and QD-NR were inducted together in the ECD assembly, as witnessed in **Fig. S6d,h**. It is inferred that Fc and CeO_2 simultaneously facilitate anodic and cathodic side reactions for BzV chromophores, which in turn render enhanced optical contrast and remarkable electrochemistry. In contrast, the ECDs utilizing bare Fc or CeO_2 do not reach their full bleaching and colored states, respectively, which in turn generates traces of irreversible layers on the electrode surface due to the dimerization tendency of BzV radical cation [3,4]. Such accumulation/deposition on the electrode surface greatly hinders the device kinetics and mounts higher charge transfer resistance on the electrode surface, thus leading to the gradual degradation of ECDs in the long term.

S1.5 Investigation of the heat effect and role of the safe potential window in the switched ECDs

Some experiments were designed and performed to analyze the extent of heat effects originating due to electrochromic switching for our flagship QD-NR/PVDF-HFP/IL/BzV-Fc ECD. The temperature distribution over the device's active area was recorded before and after 10,000 switching cycles using a thermal imaging camera (Fluke TiS75+), and the corresponding images are shown in **Fig. S7a-c**. It was observed from the thermal images that a temperature (°F in image scale) difference of ~1.1 ^{0}C persisted between the bleached and colored states of ECD (**Fig. S7a,b)**, whereas a difference of ~1.6 ^{0}C was recorded between pristine and long switched ECD (**Fig. S7a,c)**.

Considering the adequate thermal stability of gel-polymer electrolyte-based ECDs, the possibility of bubble/liquid formations due to such miniature temperature difference is rare. Further, inheriting the concern of electrolyte/PC escape, the drilled holes were sealed right after the electrolyte injection in our fabricated ECDs. Also, the blank ECD was carefully edge encapsulated through a Surlyn® frame to avoid side leakage, as mentioned in experimental **section 2.4** of the main text. In addition to the above two precautions, the presence of nanofiber assembly (PVDF-HFP) further reduces the possibility of electrolyte escape by facilitating an efficient electrolyte entrapment. Evidently, no bubble/void formation in the ECD's active area was seen after 10,000 cycles when shuttled in a voltage window of 0.9 V (0.0 to 0.9 V), as can be seen in **Fig. S7d**.

To examine the possibility of bubble formations, a duplicate QD-NR/PVDF-HFP/IL/BzV-Fc ECD was switched in an unsafe potential window of 4.0 V (-2.0 to 2.0 V) for 100 cycles. After some initial cycling, a considerable bubble/void was observed inside the ECD's active area, as shown in **Fig. S7e**. Regardless of the fact that the utilized species in the ECD were vacuumed-dried before use, the possibility of unremoved moisture/trapped air persists, for example, due to the presence of $BMIMBF_4$ (moisture prone) in the ECD. Such presence of moisture entrapment might lead to undesired reactions (such as water splitting) when subjected to a higher voltage window (here, 4.0 V), thus evolving traces of oxygen/hydrogen, which in turn might generate bubbles/voids in the ECD. Another possibility of such bubble formation results from the breakage of electrolyte/PC in such a higher operating voltage window. In short, our experimentation suggested that miniature temperature differences in ECD assembly certainly occur after numerous cycles. However, the difference is insufficient to trigger any bubble formations or electrolyte escape. On the other hand, operating ECDs in an unsafe potential window can abruptly lead to electrolyte breakage or the creation of bubbles that deters the ECD performance.

S1.6 Discussion regarding interfacial junctions and defects in hybrid nanostructures

Though quantum dots possess interesting electronic and optical properties, their high self-aggregation tendency makes it difficult to exploit their surface area fully. [5,6] Growing QDs *in-situ* over the NR surfaces was presumed to mitigate the agglomeration issue of QDs, thus retrieving more surfaces for interactions. The improved electrochromic features revealed by QD-NR-based ECD can partially be attributed to this "QD's surface recovery" aspect. In addition, the facet/surface engineering of CeO_2 is expected to tune the kinetic parameters responsible for the improved charge transfer and proliferated redox centers. [7,8] It is now in good agreement that distinct morphologies in CeO_2 nanostructure deliver dissimilar degrees of surface electronic structures, reaction centers, electric conductivity, and adsorption sites depending on the exposed facets. [9–12] The interesting feature of 0D/1D hybrid nanostructures (in our study, QD-NR) can be attributed to discrete interfaces and individual components with their own crystal planes of varying orientation. Such properties enable the hybrid nanostructures to possess singly charged and neutral vacancies at the defective junctions (0D/1D). [7]

We also observed the presence of several surface vacancies in the synthesized QD-NR filler due to microstrains, as witnessed through XPS and EPR spectra in **Fig. 1**. The available microstrains in such tuned nanostructures are reported to have low formation energies and facilitate the creation of an anion Frankel defect, which in turn causes numerous single and surface oxygen vacancies. The synergistic shuffling of single and surface oxygen vacancies leads to considerable structural rearrangement in the crystal lattice of CeO_2, forming distinct dimeric and trimeric vacancy clusters with different orientations. [8,12] Briefly, it can be deciphered that the availability of reactive surfaces and synergistic improvement brought by defect surfaces and corrugated junctions seems to have a decisive role that allows hybrid nanostructures such as QD-NR in our study to deliver superior electrochromic performance as compared to their bare NR and QD counterparts.

S2. Table and Figures

Table S1. Quantification of redox centers for as-synthesized nanofillers

	CeO_2 nanofillers		
	**NR**	**QD**	**QD-NR**
**[Ce^{4+}] %**	73.3	80.3	67.8
**[Ce^{3+}] %**	26.7	19.7	32.2

Table S2. Optical contrast of ECDs with different filler loadings

Device	CeO_2 NR wt%	ΔT (%), 603 nm
PVDF-HFP/IL/BzV-Fc ECD	0.0	57.3
$NR_{0.5}$/PVDF-HFP/IL/BzV-Fc ECD	0.5	56.0
$NR_{1.0}$/PVDF-HFP/IL/BzV-Fc ECD	1.0	60.1
$NR_{1.5}$/PVDF-HFP/IL/BzV-Fc ECD	1.5	58.7
$NR_{2.0}$/PVDF-HFP/IL/BzV-Fc ECD	2.0	53.1

Table S3. Electrolyte uptake (EU) and volumetric porosity (VP) of as-spun membranes

Membrane species	EU (%)	VP (%)
PVDF-HFP/IL	636.7 ± 16.1	81.6 ± 1.3
NR/PVDF-HFP/IL	515.8 ± 13.4	75.3 ± 0.7
QD/PVDF-HFP/IL	522.9 ± 14.0	76.9 ± 1.1
QD-NR/PVDF-HFP/IL	578.1 ± 18.7	79.1 ± 1.5

Table S4. Transmittance and stability analysis of host PVDF-HFP/IL ECD with different configurations

PVDF-HFP/IL/X ECD	T_c (%)	T_b(%)	ΔT	Stability (1000 cycles)
BzV	75.2	19.3	55.9	Poor
BzV-Fc	76.3	19.1	57.2	Marginal
QD-NR/BzV	84.6	26.2	58.4	Marginal
QD-NR/BzV-Fc	85.1	17.3	67.8	Excellent

Table S5. Partial list of recent reports announcing major developments in the field of SPE/GPE based ECDs

ECD Configuration	Operating window (V)	λ_{max} (nm)	ΔT (%)	τ_c (s)/τ_b(s)	η_{col} (cm^2/C)	Cycling stability (ΔT-retention)	Ref.
ITO/poly-(PC)+gel electrolyte+ PEDOT/ITO	−0.9 to 1.5 = 2.4 V	650	~28	0.5/0.4	~981	92% after 5000 cycles	[13]
ITO/ PANI-TiO_2+P(VDF-HFP) +15 wt% taurine+ $BMIMBF_4$/ITO	+2.0 to −2.0 = 4.0 V	650	~56	2.0/2.5	-	-	[14]
ITO/ $NV(BF_4)_2$+ 20 wt% PIL+Fc/ITO	0 to 1.2 = 1.2 V	605	~55	2.1/2.1	~273	~97% after 10,000 cycles	[15]
ITO/ PTMA-*co*-BP+ECP magenta + (EMI-TFSI) +PC/ITO	+1.2 to −1.35 = 2.55 V	550	~72	4.6/9.5	~754	~84% after 1800 cycles	[16]
ITO/Sb-doped SnO_2+ TiO_2 + viologen/ITO	0 to -1.5 = 1.5 V	605	~58	0.5/0.6	~440	~83% after 500 cycles	[17]
FTO/PNIPAm+ PEDOT/FTO**	−0.4 to 0.4 = 0.8 V	550	~26	0.9/0.6	-	>90 % after 200 cycles	[18]
ITO/PEG+ ACN+ PI-2a/ITO**	0 to 1.2 =1.2 V	760	~96	2.2/3.7	~78	~79% (5000 cycles)	[19]
ITO/PET/EV+dmFc+ ChCl+MPG-eutectogel/ ITO/PET *(flexible ECD)*	-0.7 to 0 =0.7 V	605	~59	~15/~30	-	~94% after 100 cycles	[20]
ITO/ PVDF-HFP+ $BMIMBF_4$+ (OHV-POSS)+(IO-POSS)+mFc/ITO	0 to −2 =2.0 V	532	~67	8.6/8.8	~308	~82% after 12,000 cycles	[21]
ITO/$EtV(Br)_2$+PMMA-r-PBA+EMI-TFSI+ dmFc/ITO	0 to 0.8 =0.8 V	601	~97	10.1/ 50.7	~89	-	[22]

FTO/ P-WO_3 +PIL B /FTO**	2.0 ~1.5 =3.5 V	660	~56	1.7/6.4	-	~84% (5000 cycles)	[23]
PET/PProDOT+ gel-electrolyte +PEDOT: PSS-AgNWs/PET *(flexible ECD)*	−0.9 to 0.9 =1.8 V	545	~59	7.4/3.9	~424	~86 % after 1000 cycles	[24]
ITO/QD-NR +PVDF-HFP+IL + BzV+Fc/ITO	0 to 0.9 =0.9 V	603	~69	1.9/2.2	~339	~90% after 25000 cycles	**This work**

λ_{max}: maximum wavelength, ΔT: Transmittance change, τ_c, and τ_b: coloration and bleaching time, η_{col}: Coloration efficiency.
***Only the best-performing ECD configuration is chosen for comparison.*

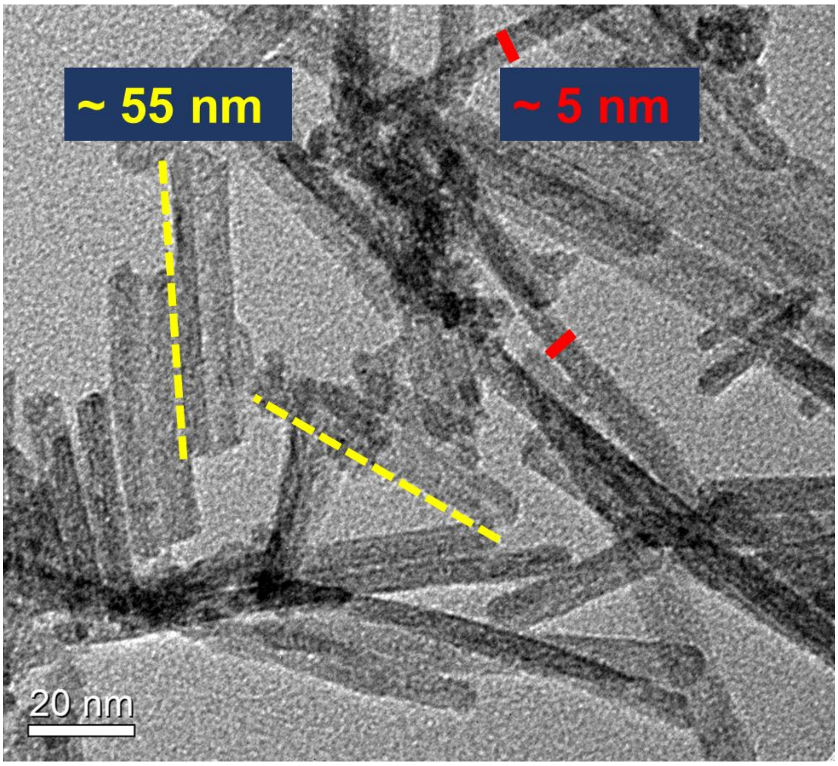

Fig. S1. The TEM image highlights the average length and width of synthesized CeO_2 nanorods.

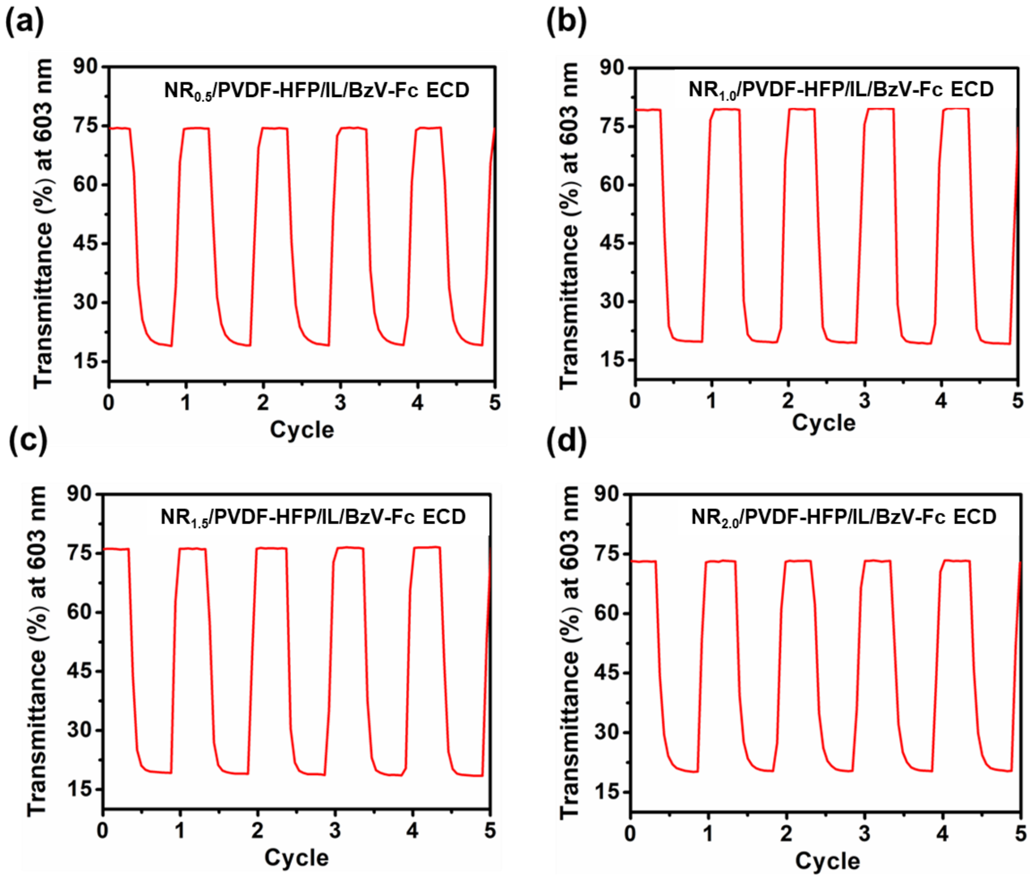

Fig. S2. The transmittance attenuation of NR/PVDF-HFP/IL/BzV-Fc ECD at 603 nm switched between 0 and 1.0 V at a different filler loading of **(a)** 0.5, **(b)** 1.0, **(c)** 1.5, and **(d)** 2.0 wt%.

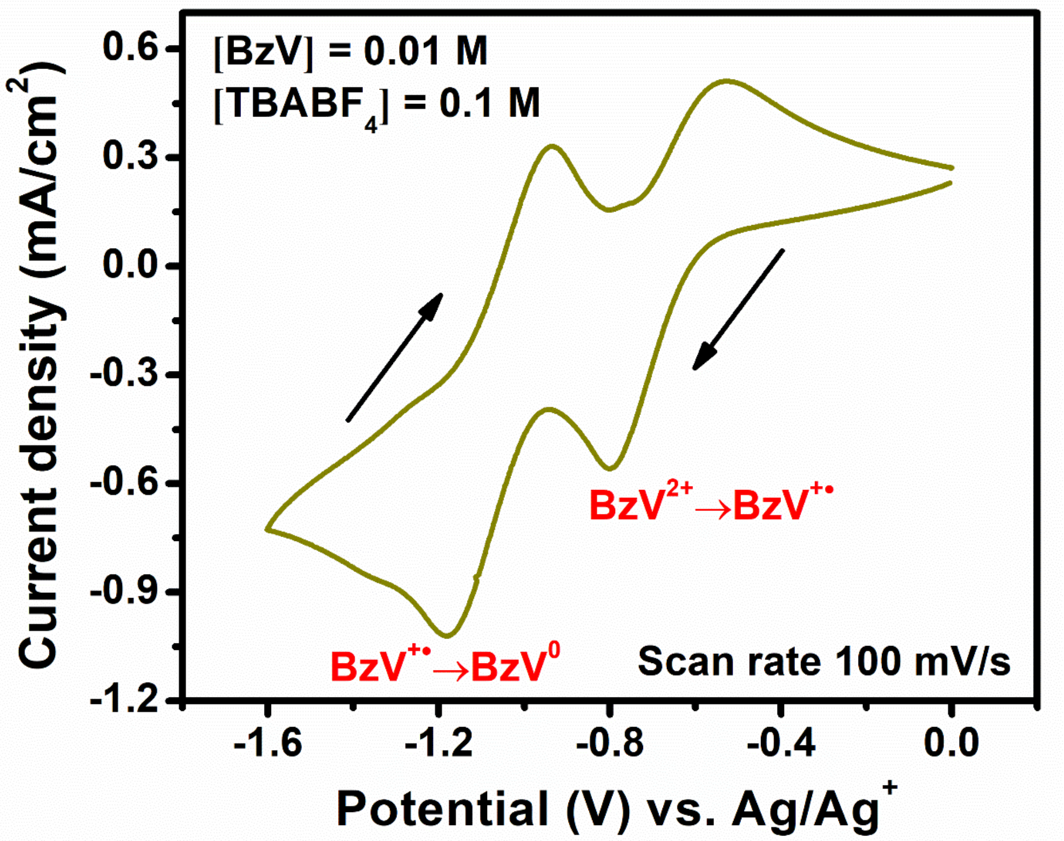

Fig. S3. The cyclic voltammogram of electrochromic benzyl viologen (BzV) in a three-electrode system utilizing $TBABF_4$ as a supporting electrolyte, platinum wire as a counter, and Ag/Ag^+ as a reference electrode.

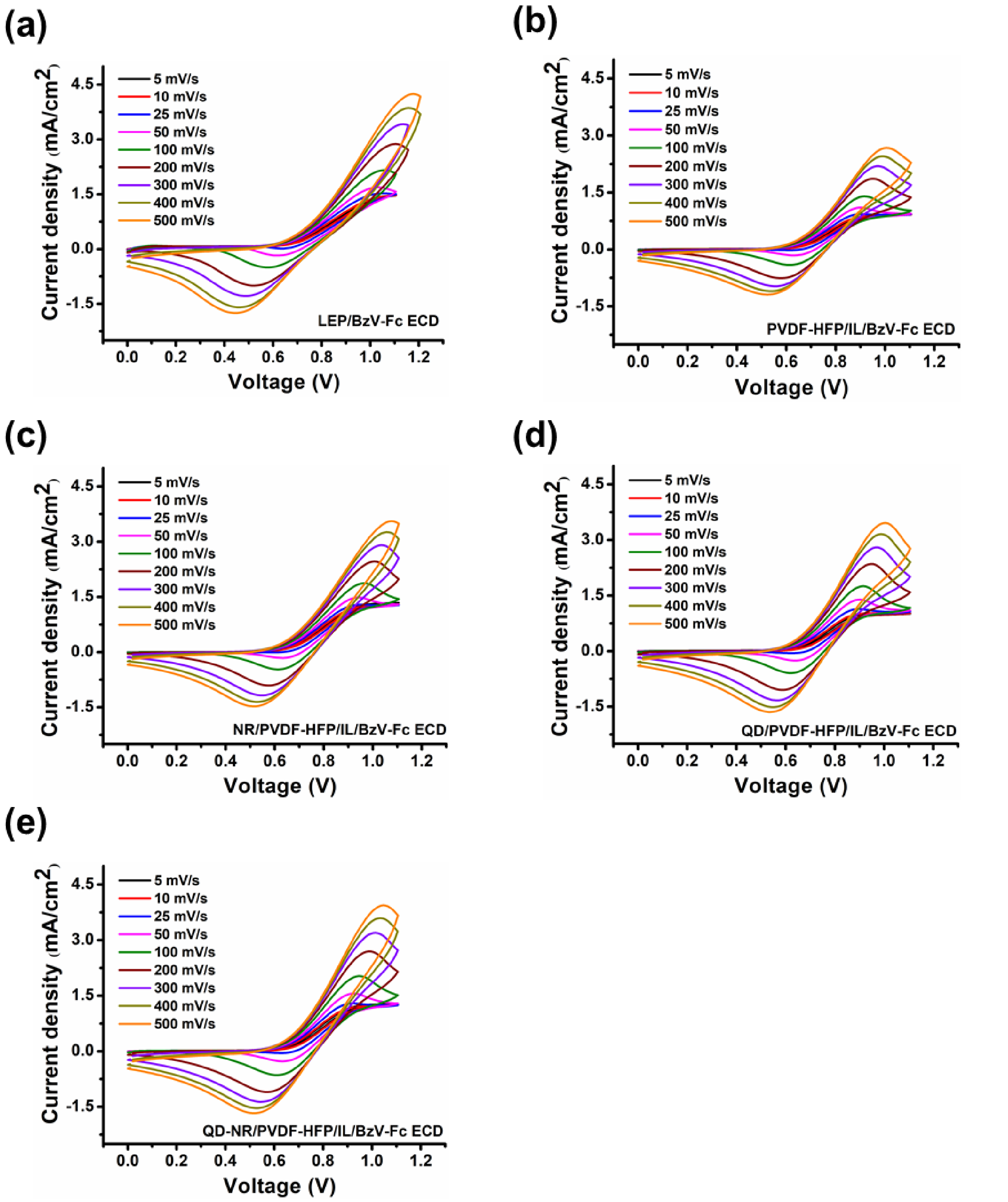

Fig. S4. The cyclic voltammograms of **(a)** LEP/BzV-Fc ECD**, (b)** PVDF-HFP/IL/BzV-Fc ECD, **(c)** NR/PVDF-HFP/IL/BzV-Fc ECD, **(d)** QD/PVDF-HFP/IL/BzV-Fc ECD, and **(e)** QD-NR/PVDF-HFP/IL/BzV-Fc ECD employed under incremental scan rate of 5 ~ 500 mV/s in a voltage window between 0 and 1.2 V.

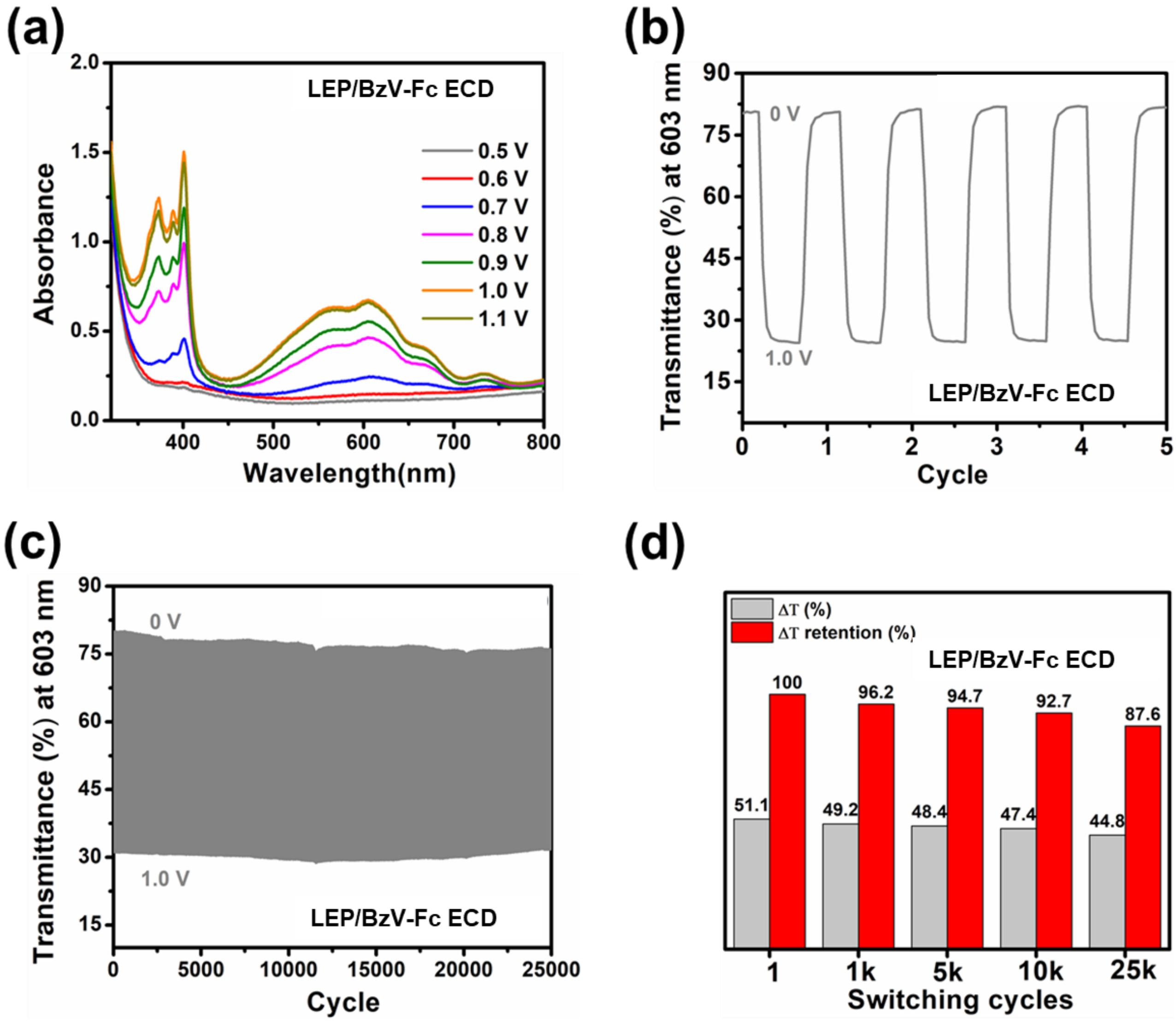

Fig. S5. (a) The UV-Vis absorbance spectra for LEP/BzV-Fc ECD switched from 0 to 1.1 V with a step increment of 0.1 V. **(b)** The transmittance spectra, **(c)** switching stability plots for 25,000 cycles, and **(d)** retention analysis data for an LEP/BzV-Fc ECD switched between 0 and 1.0 V at 603 nm.

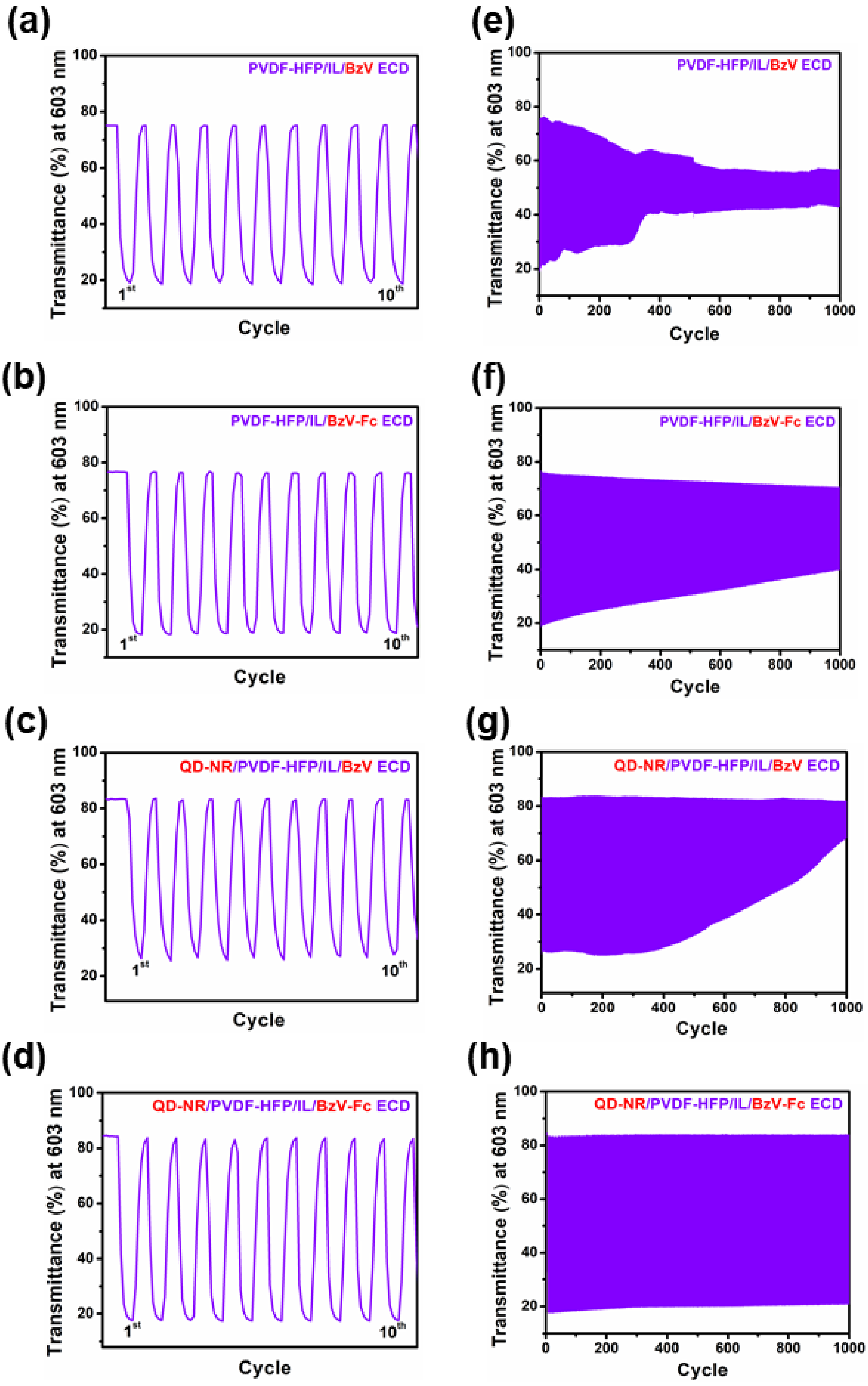

Fig. S6. The dynamic transmittance and stability plots of host PVDF-HFP/IL ECD with **(a,e)** bare BzV, **(b,f)** BzV-Fc, **(c,g)** QD-NR/BzV and **(d,h)** QD-NR/BzV-Fc configurations.

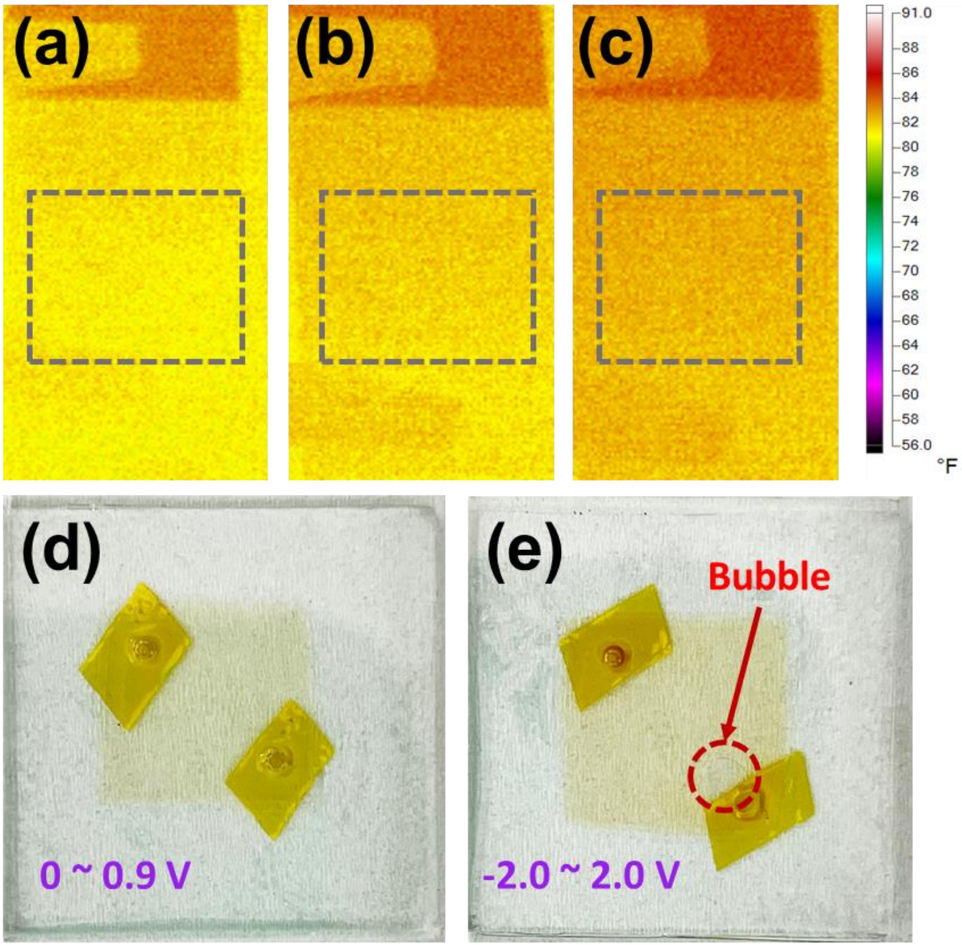

Fig. S7. Thermal imaging (IR) of QD-NR/PVDF-HFP/IL/BzV-Fc ECD during **(a)** bleaching, **(b)** coloration, and **(c)** after completion of 10,000 switching cycles. Visuals of QD-NR/PVDF-HFP/IL/BzV-Fc ECD with switching cycles/voltage window (V) of **(d)** 10,000/0.9 V and **(e)** 100/4.0 V

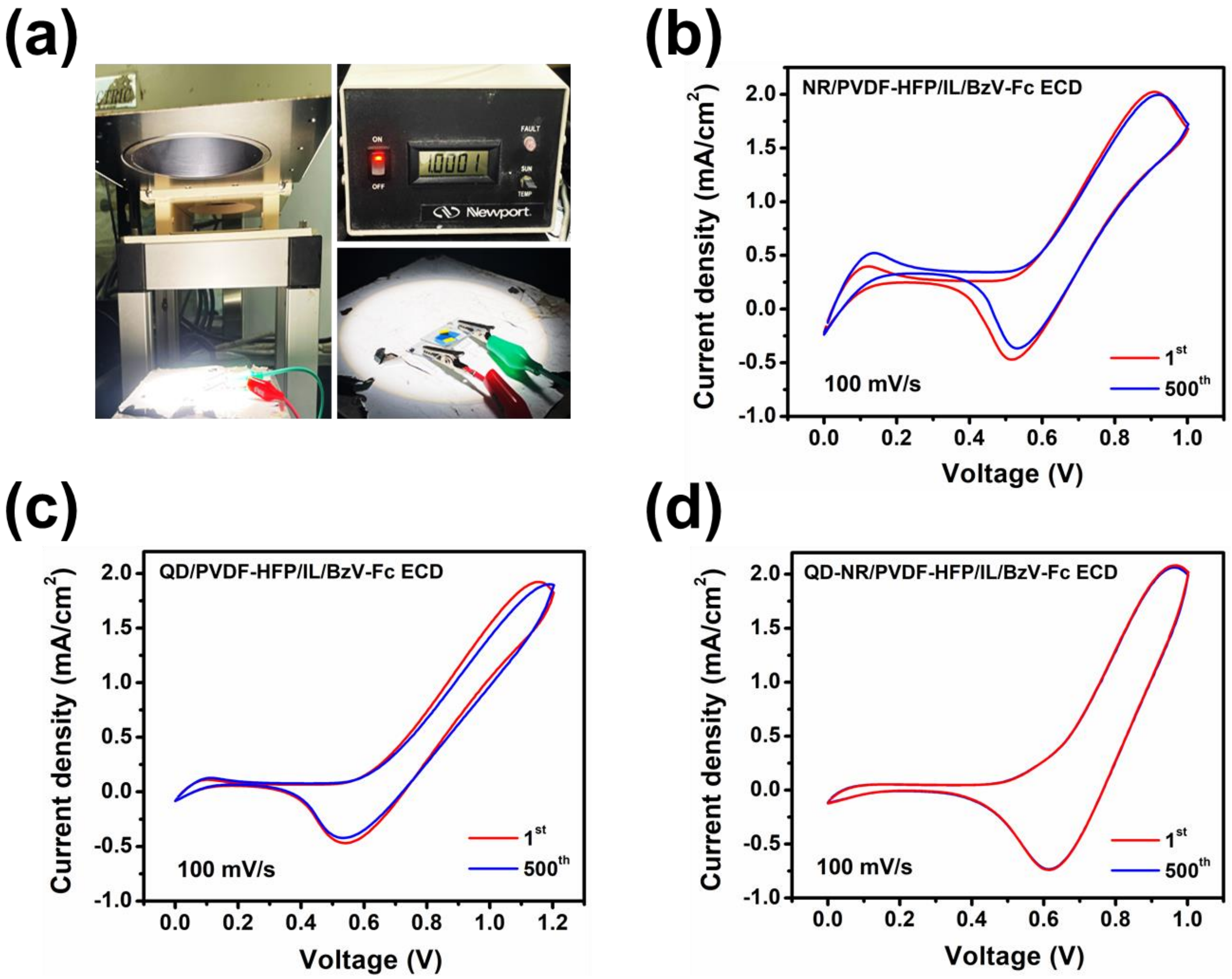

Fig. S8. (a) Visuals of the setup utilized for solar irradiation. The CV plots for the **(b)** NR/PVDF-HFP/IL/BzV-Fc ECD, **(c)** QD/PVDF-HFP/IL/BzV-Fc ECD, and **(d)** QD-NR/PVDF-HFP/IL/BzV-Fc ECD illuminated at 1 sun (100 mW cm^{-2}, AM 1.5G) condition.

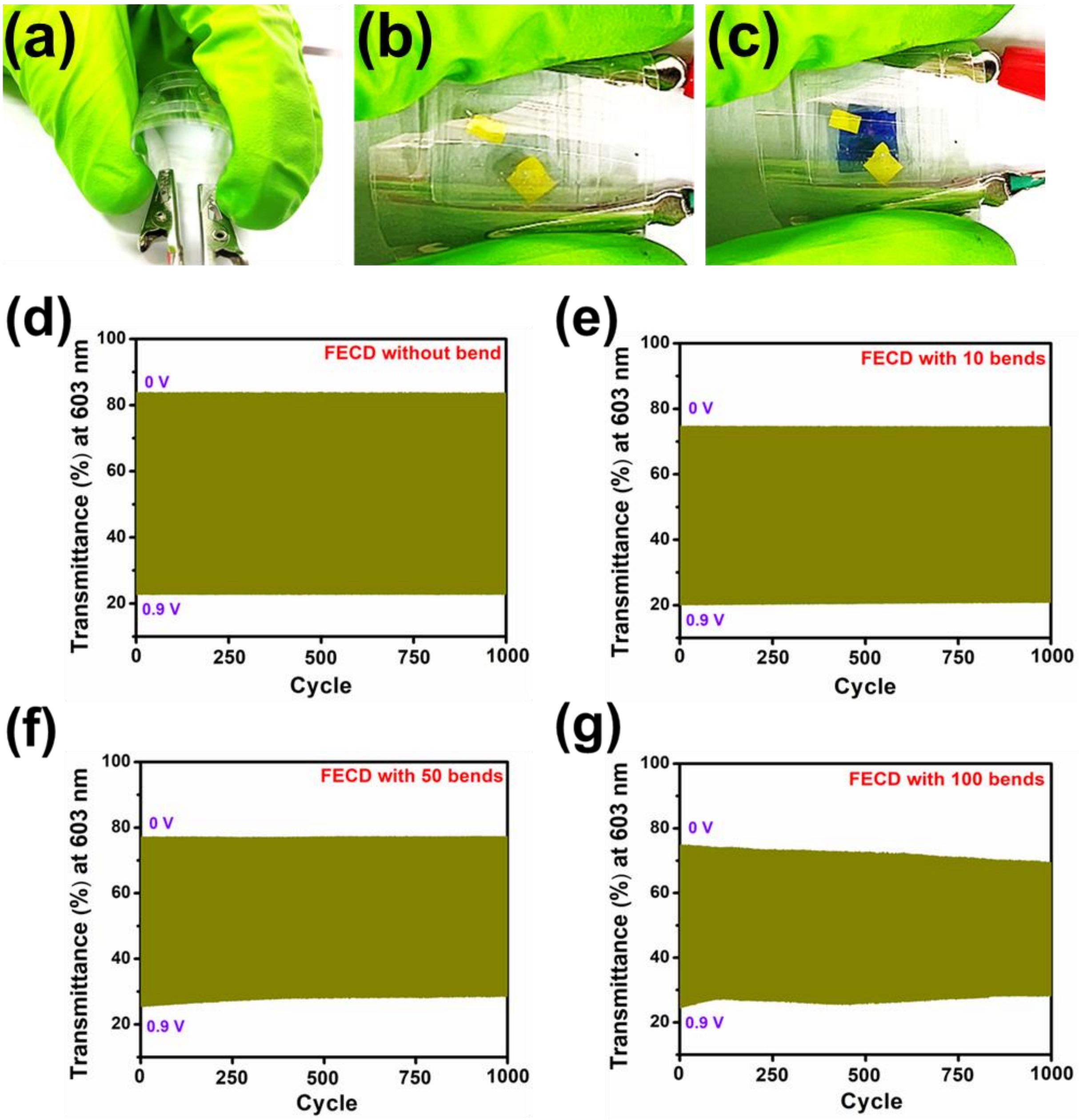

Fig. S9. (a) Visuals of the flexible ECD (QD-NR/PVDF-HFP/IL/BzV-Fc FECD) in **(b)** bleached and **(c)** colored state. (**d)** The switching stability plots of the as-prepared flexible ECD. The switching stability plots of the flexible ECD after **(e)** 10, **(f)** 50, and **(g)** 100 bends.

References:

[1] H.C. Lu, S.Y. Kao, H.F. Yu, T.H. Chang, C.W. Kung, K.C. Ho, Achieving Low-Energy Driven Viologens-Based Electrochromic Devices Utilizing Polymeric Ionic Liquids, ACS Appl. Mater. Interfaces. 8 (2016) 30351–30361. https://doi.org/10.1021/acsami.6b10152.

[2] H.F. Yu, K.I. Chen, M.H. Yeh, K.C. Ho, Effect of trifluoromethyl substituents in benzyl-based viologen on the electrochromic performance: Optical contrast and stability, Sol. Energy Mater. Sol. Cells. 200 (2019) 110020. https://doi.org/10.1016/j.solmat.2019.110020.

[3] Y. Alesanco, A. Viñuales, G. Cabañero, J. Rodriguez, R. Tena-Zaera, Colorless to Neutral Color Electrochromic Devices Based on Asymmetric Viologens, ACS Appl. Mater. Interfaces. 8 (2016) 29619–29627. https://doi.org/10.1021/acsami.6b11321.

[4] R. Sydam, M. Ojha, M. Deepa, Ionic additive in an ionogel for a large area long lived high contrast electrochromic device, Sol. Energy Mater. Sol. Cells. 220 (2021) 110835. https://doi.org/https://doi.org/10.1016/j.solmat.2020.110835.

[5] Q. Xiong, S. Huang, J. Du, X. Tang, F. Zeng, Z. Liu, Z. Zhang, T. Shi, J. Yang, D. Wu, H. Lin, Z. Luo, Y. Leng, Surface Ligand Engineering for CsPbBr3 Quantum Dots Aiming at Aggregation Suppression and Amplified Spontaneous Emission Improvement, Adv. Opt. Mater. 8 (2020) 2000977. https://doi.org/https://doi.org/10.1002/adom.202000977.

[6] S. Chen, J.-W. Liu, M.-L. Chen, X.-W. Chen, J.-H. Wang, Unusual emission transformation of graphene quantum dots induced by self-assembled aggregation, Chem. Commun. 48 (2012) 7637–7639. https://doi.org/10.1039/C2CC32984K.

[7] R.C. de Oliveira, R.A.C. Amoresi, N.L. Marana, M.A. Zaghete, M. Ponce, A.J. Chiquito, J.R. Sambrano, E. Longo, A.Z. Simões, Influence of Synthesis Time on the Morphology and Properties of CeO2 Nanoparticles: An Experimental–Theoretical Study, Cryst. Growth Des. 20 (2020) 5031–5042. https://doi.org/10.1021/acs.cgd.0c00165.

[8] S. Sultana, S. Mansingh, K.M. Parida, Crystal facet and surface defect engineered low dimensional CeO2 (0D, 1D, 2D) based photocatalytic materials towards energy generation and pollution abatement, Mater. Adv. 2 (2021) 6942–6983. https://doi.org/10.1039/D1MA00539A.

[9] K. Zhao, J. Qi, H. Yin, Z. Wang, S. Zhao, X. Ma, J. Wan, L. Chang, Y. Gao, R. Yu, Z. Tang, Efficient water oxidation under visible light by tuning surface defects on ceria nanorods, J. Mater. Chem. A. 3 (2015) 20465–20470. https://doi.org/10.1039/C5TA05817A.

[10] R.A.C. Amoresi, R.C. Oliveira, N.L. Marana, P.B. de Almeida, P.S. Prata, M.A. Zaghete, E. Longo, J.R. Sambrano, A.Z. Simões, CeO2 Nanoparticle Morphologies and Their Corresponding Crystalline Planes for the Photocatalytic Degradation of Organic Pollutants, ACS Appl. Nano Mater. 2 (2019) 6513–6526. https://doi.org/10.1021/acsanm.9b01452.

[11] S.C. Kuiry, S.D. Patil, S. Deshpande, S. Seal, Spontaneous Self-Assembly of Cerium Oxide Nanoparticles to Nanorods through Supraaggregate Formation, J. Phys. Chem. B. 109 (2005) 6936–6939. https://doi.org/10.1021/jp050675u.

[12] B. Huang, R. Gillen, J. Robertson, Study of CeO2 and Its Native Defects by Density Functional Theory with Repulsive Potential, J. Phys. Chem. C. 118 (2014) 24248–24256. https://doi.org/10.1021/jp506625h.

[13] M. Sezgin, O. Ozay, S. Koyuncu, H. Ozay, F. Baycan Koyuncu, A neutral state colorless phosphazene/carbazole hybride dendron and its electrochromic device application, Chem. Eng.

J. 274 (2015) 282–289. https://doi.org/https://doi.org/10.1016/j.cej.2015.03.134.

[14] R. Zhou, W. Liu, Y.W. Leong, J. Xu, X. Lu, Sulfonic Acid- and Lithium Sulfonate-Grafted Poly(Vinylidene Fluoride) Electrospun Mats As Ionic Liquid Host for Electrochromic Device and Lithium-Ion Battery, ACS Appl. Mater. Interfaces. 7 (2015) 16548–16557. https://doi.org/10.1021/acsami.5b04034.

[15] H.-C. Lu, S.-Y. Kao, H.-F. Yu, T.-H. Chang, C.-W. Kung, K.-C. Ho, Achieving Low-Energy Driven Viologens-Based Electrochromic Devices Utilizing Polymeric Ionic Liquids, ACS Appl. Mater. Interfaces. 8 (2016) 30351–30361. https://doi.org/10.1021/acsami.6b10152.

[16] J. He, S. Mukherjee, X. Zhu, L. You, B.W. Boudouris, J. Mei, Highly Transparent Crosslinkable Radical Copolymer Thin Film as the Ion Storage Layer in Organic Electrochromic Devices, ACS Appl. Mater. Interfaces. 10 (2018) 18956–18963. https://doi.org/10.1021/acsami.8b03235.

[17] C. Kortz, A. Hein, M. Ciobanu, L. Walder, E. Oesterschulze, Complementary hybrid electrodes for high contrast electrochromic devices with fast response, Nat. Commun. 10 (2019) 4874. https://doi.org/10.1038/s41467-019-12617-4.

[18] M. Wang, X. Xing, I.F. Perepichka, Y. Shi, D. Zhou, P. Wu, H. Meng, Electrochromic Smart Windows Can Achieve an Absolute Private State through Thermochromically Engineered Electrolyte, Adv. Energy Mater. 9 (2019) 1900433. https://doi.org/https://doi.org/10.1002/aenm.201900433.

[19] Q. Zhang, C.-Y. Tsai, L.-J. Li, D.-J. Liaw, Colorless-to-colorful switching electrochromic polyimides with very high contrast ratio, Nat. Commun. 10 (2019) 1239. https://doi.org/10.1038/s41467-019-09054-8.

[20] C. Gu, Y. Peng, J. Li, H. Wang, X.-Q. Xie, X. Cao, C.-S. Liu, Supramolecular G4 Eutectogels of Guanosine with Solvent-Induced Chiral Inversion and Excellent Electrochromic Activity, Angew. Chemie Int. Ed. 59 (2020) 18768–18773. https://doi.org/https://doi.org/10.1002/anie.202009332.

[21] G.K. Pande, J.H. Choi, J.-E. Lee, Y.E. Kim, J.H. Choi, H.W. Choi, H.G. Chae, J.S. Park, Octa-viologen substituted polyhedral oligomeric silsesquioxane exhibiting outstanding electrochromic performances, Chem. Eng. J. 393 (2020) 124690. https://doi.org/https://doi.org/10.1016/j.cej.2020.124690.

[22] S.Y. Kim, Y.J. Jang, Y.M. Kim, J.K. Lee, H.C. Moon, Tailoring Diffusion Dynamics in Energy Storage Ionic Conductors for High-Performance, Multi-Function, Single-Layer Electrochromic Supercapacitors, Adv. Funct. Mater. 32 (2022) 2200757. https://doi.org/https://doi.org/10.1002/adfm.202200757.

[23] W.C. Poh, A.L.-S. Eh, W. Wu, X. Guo, P.S. Lee, Rapidly Photocurable Solid-State Poly(ionic liquid) Ionogels For Thermally Robust and Flexible Electrochromic Devices, Adv. Mater. 34 (2022) 2206952. https://doi.org/https://doi.org/10.1002/adma.202206952.

[24] X. Lv, H. Xu, Y. Yang, M. Ouyang, M. Xia, C. Liu, D.S. Wright, C. Zhang, Flexible laterally-configured electrochromic supercapacitor with feasible patterned display, Chem. Eng. J. 458 (2023) 141453. https://doi.org/https://doi.org/10.1016/j.cej.2023.141453.
